# The transcription factor RUNT-like regulates pupal cuticle development via promoting a pupal cuticle protein transcription

**DOI:** 10.1371/journal.pgen.1011393

**Published:** 2024-09-12

**Authors:** Ke-Yan Jin, Xiao-Pei Wang, Yu-Qin Di, Yu-Meng Zhao, Jin-Xing Wang, Xiao-Fan Zhao

**Affiliations:** Shandong Provincial Key Laboratory of Animal Cells and Developmental Biology, School of Life Sciences, Shandong University, Qingdao, China; University of Kentucky, UNITED STATES OF AMERICA

## Abstract

Holometabolous insects undergo morphological remodeling from larvae to pupae and to adults with typical changes in the cuticle; however, the mechanism is unclear. Using the lepidopteran agricultural insect *Helicoverpa armigera*, cotton bollworm, as a model, we revealed that the transcription factor RUNT-like (encoded by *Runt-like*) regulates the development of the pupal cuticle via promoting a pupal cuticle protein gene (*HaPcp*) expression. The *HaPcp* was highly expressed in the epidermis and wing during metamorphosis and was found being involved in pupal cuticle development by RNA interference (RNAi) analysis in larvae. *Runt-like* was also strongly upregulated in the epidermis and wing during metamorphosis. Knockdown of *Runt-like* produced similar phenomena, a failure of abdomen yellow envelope and wing formation, to those following *HaPcp* knockdown. The insect molting hormone 20-hydroxyecdysonen (20E) upregulated *HaPcp* transcription via RUNT-like. 20E upregulated *Runt-like* transcription via nuclear receptor EcR and the transcription factor FOXO. Together, RUNT-like and HaPCP are involved in pupal cuticle development during metamorphosis under 20E regulation.

## Author summary

Holometabolous insects develop pupal cuticle during metamorphosis; however, the mechanism is unclear. Using the lepidopteran agricultural insect *Helicoverpa armigera*, cotton bollworm, as a model, we revealed that the transcription factor RUNT-like regulates a pupal cuticle protein gene, *HaPcp*, expression therefore regulates the development of the pupal cuticle under insect molting hormone 20-hydroxyecdysonen (20E) regulation. *HaPcp* is an insect specific gene without homologue in humans, and the insect *Runt-like* is different from vertebrate, too. This work deeply addresses the mechanism that 20E regulates development of insect pupal cuticle and presents new gene targets to explore the new growth regulators to control the pest.

## Introduction

Holometabolous insects, such as the lepidopteran insect *Helicoverpa armigera*, cotton bollworm, show tissue remodeling during metamorphosis under regulation of the molting hormone 20-hydroxyecdysonen (20E), including remodeling of the midgut and fat body by degradation of larval tissue and development of adult tissue [1]. The morphology of the cuticle also changes greatly from larva to pupa. However, the mechanism of remodeling of cuticle is not fully understood.

The integument of insects consists of the cuticle and epidermis from outer to inner, and the cuticle is further divided into the envelope, epicuticle, exocuticle and endocuticle from outside to inside [2]. The epidermis is the single layer of live cells that secrete lipids, chitin and cuticle proteins to form the noncell cuticle [3]. Cuticle proteins are the main component of the integument and wings. For example, the *Locusta migratoria* cuticle protein LmACP7 is an essential structural protein in wings [4]. In *Bombyx mori*, the cuticle protein BmCPG4 keeps the hind wings normal [5]. Wing disc cuticle proteins (WCPs) are specifically expressed in the epidermis and are responsible for the formation of the pupal cuticle during the transition from larva to pupa. *B*. *mori* BmWCP4 promotes pupal cuticle formation, and BmWCP5 promotes wing primordia development [6].

The wing of holometabolous insects is developed from the wing disc, which is also derived from the ectoderm as the epidermis. Wing is regulated by growth signals and completes development in the pupal stage [7]. The wing primordium of dipteran *Drosophila* is composed of a single layer of epithelial cells, which form the dorsal and ventral surfaces of adult wings by folding and everting during growth and development, while the wing primordium of Lepidoptera is composed of two layers of epithelial cells that form the dorsal and ventral surfaces of adult wings through growth and development, respectively [8].

20E is the major hormone that promotes insect metamorphosis [9] and is increased during metamorphosis in *H*. *armigera* [10]. 20E regulates the expression of metamorphic stage-specific transcription factors through the nuclear receptors EcR and USP [11]. For example, 20E upregulates the expression of the typical metamorphic stage-specific transcription factor Foxhead box protein O (FOXO), which further regulates the expression of some downstream target genes, such as *Br* and *Hr3* [12]. The expression of some epidermal proteins is metamorphic stage-specific and is the regulatory target of transcription factors and 20E [13]. A study in *Drosophila melanogaster* found that 20E activates the expression of the pupal cuticle protein-encoding gene *Edg84a* by activating the transcription factor *β-Ftz-F1* [14]. 20E upregulates the expression of the oncogene *c-Myc* within one hour, promotes the expression of *Cyclins* and promotes wing development [15]. However, other mechanisms by which 20E regulates cuticle remodeling and wing development remain unclear.

RUNT is an important transcription factor in Runt-related transcription factor (RUNX) family, playing roles in cell proliferation, differentiation and maintenance of stem cells [16]. According to literature reports, the first *Runt* gene is discovered in *D*. *melanogaster* [17]. The *Runx* gene is later found in all metazoans [18]. All members of the RUNX family have a highly conserved Runt domain at the N-terminus, which is needed for heterodimerization and DNA binding [19]. RUNT domain in RUNX is for heterodimerization and DNA binding, and forms as an immunoglobulin fold, which is shared by P53, NF-kB, STAT, RNUX1, X2, X3 in mammals [20]. There are three *Runx* genes in mammals: RUNX1 is needed for decisive hematopoiesis and is a frequently mutated gene in human leukemia; RUNX2 is needed for osteogenesis and is associated with clavicular cranial dysplasia; and RUNX3 controls dorsal root ganglion neurogenesis and gastric epithelial cell proliferation [21]. Studies in *B*. *mori* showed that *BmRunt* is only expressed in the ovaries on day 3 of 5th instar larvae, suggesting that it is associated with oogenesis, and no *BmRunt* transcripts are detected in any other tissues [22]. In *D*. *melanogaster*, RUNT and Lozenge (*Lz*, another RUNX family protein gene in *D*. *melanogaster*) regulate a large number of developmental processes [23], and DmRUNT plays a key role in embryonic neural development [17, 23]. However, it is unclear whether RUNT regulates the development of the insect cuticle.

In this study, we found that the pupal cuticle protein gene *HaPcp*, and transcription factor *Runt-like* are highly expressed in the epidermis and wing during metamorphosis. HaPCP participated in the formation of the pupal cuticle. RUNT-like promotes the transcription of *HaPcp*, and 20E promotes the expression of *Runt-like* through its nuclear receptor EcR and transcription factor FOXO, thus, 20E promotes the development of the pupal cuticle.

## Results

### Pupal cuticle proteins were highly expressed during metamorphosis

**Fig 1:**
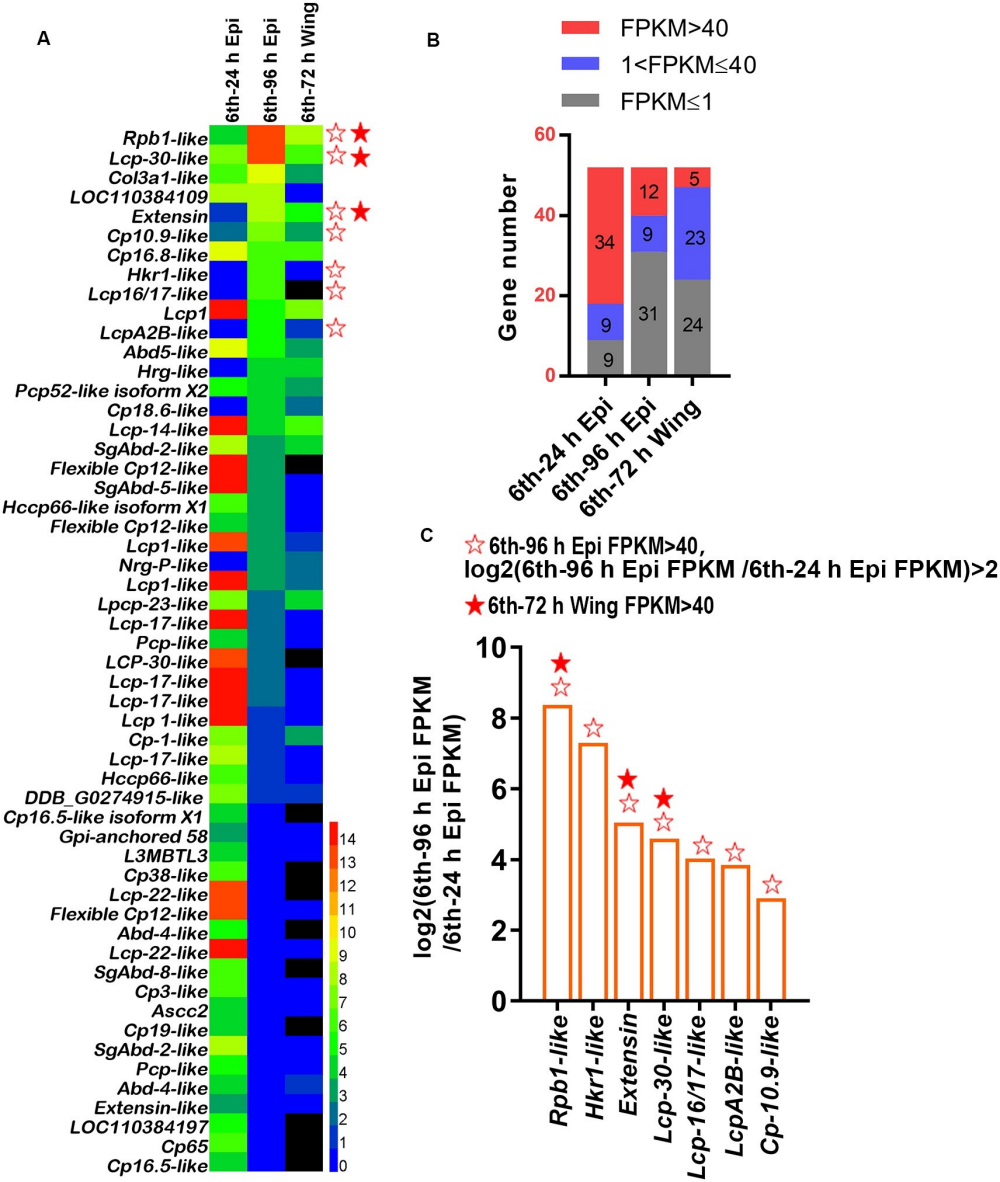
Expression analysis and screening of cuticle proteins expressed in the epidermis and wings. (A) Hemi heatmap to analyze the expression of all cuticle proteins in the 6th-24 h epidermis, 6th-96 h epidermis and 6th-72 h wing. (B) FPKM value analysis of cuticle proteins in the 6th-24 h epidermis, 6th-96 h epidermis and 6th-72 h wing. (C) Cuticle proteins with upregulated expression in the 6th-96 h epidermis and FPKM values greater than 40 in the 6th-96 h epidermis and 6th-72 h wings. The vertical axis represents the value of log2 (6th-96 h Epi FPKM/6th-24 h Epi FPKM). The hollow stars represent seven cuticle proteins that were upregulated in the 6th-96 h epidermis and had FPKM values greater than 40. The solid stars represent the cuticle proteins with FPKM values greater than 40 in the 6th-72 h wing of the seven cuticle proteins.

*H*. *armigera* has six larval instars. The metamorphosis begins from the last larval instar (6th larval instar 72 h), goes through wandering stages, then pupation at about 6th larval instar 140 h. The adult wings gradually developed from 6th larval instar 48 h to pupation. To explore the molecular mechanism of cuticle remodeling during development, we identified all cuticle proteins in the epidermis and wings of *H*. *armigera* from the transcriptomes of the epidermis and wing (S1 Table). The Hemi heat map showed that the expression of the cuticle proteins is stage-specific at the 6th larval stage 24 h (6th-24 h) feeding stage and at the 6th larval stage 96 h (6th-96 h) metamorphic stage in the epidermis and at the 6th larval stage 72 h (6th-72 h) wing. Several cuticle protein genes had upregulated expression with Fragments Per Kilobase of exon model per Million mapped fragment (FPKM) values >40 in the 6th-96 h epidermis, including DNA-directed RNA polymerase II subunit RPB1-like (*Rpb1-like*, another name in the genome of *H*. *armigera* is hypothetical protein B5X24_HaOG214610 with Sequence ID: PZC80453.1), larval cuticle protein LCP-30 (*Lcp-30-like*), *Extensin* (uncharacterized protein), signaling mucin cuticle protein10.9-like (*Cp-10*.*9-like*), HKR1 (*Hkr1-like*), LCP16/17-like (*Lcp-16/17-like*), and larval cuticle protein LCPA2B-like (*LcpA2B-like*), and 3 cuticle protein genes (*Rpb1-like*, *Extensin*, *Lcp-30-like*) had FPKM values >40 in wings (Fig 1A–1C).

### RPB1-like was identified as a pupal cuticle protein HaPCP

RPB1-like of *H*. *armigera* was classified by Mega 7.0 Software using the identical proteins found from genomes of *Homo sapiens*, *H*. *armigera*, *B*. *mori*, *D*. *melanogaster* and *Manduca sexta* in the NCBI website to identify its characteristics. The phylogenetic tree showed that RPB1-like (XP 021186897.1) was clustered with the *B*. *mori* cuticle protein RR-1 and aggregated into a larger clade with other cuticle proteins. In contrast, the DNA-directed RNA polymerase II subunit RPB1 of *H*. *armigera* (XP 021188795.1) was aggregated into another branch with other species RPB1 (S1A Fig). In addition, RPB1-like of *H*. *armigera* contained a chitin-binding-4 domain, like the *B*. *mori* cuticle protein RR-1, while the RPB1 of *H*. *armigera* and other species had no chitin-binding-4 domain (S1B Fig). Further alignment of RPB1-like of *H*. *armigera* with other cuticle proteins showed that the chitin-binding domain is conserved in the cuticle proteins in lepidopteran insects, including *B. mori* cuticular protein RR-1 motif 21 isoform and *Spodoptera litura* cuticular protein RR-1 (S2 Fig). Therefore, RPB1-like of *H*. *armigera* is a pupal cuticle protein and renamed as *Helicoverpa armegera* pupal cuticle protein (HaPCP) in this work. HaPCP is also predicted to bind chitin by software SYBYL-X 2.0 (S3 Fig).

**Fig 2:**
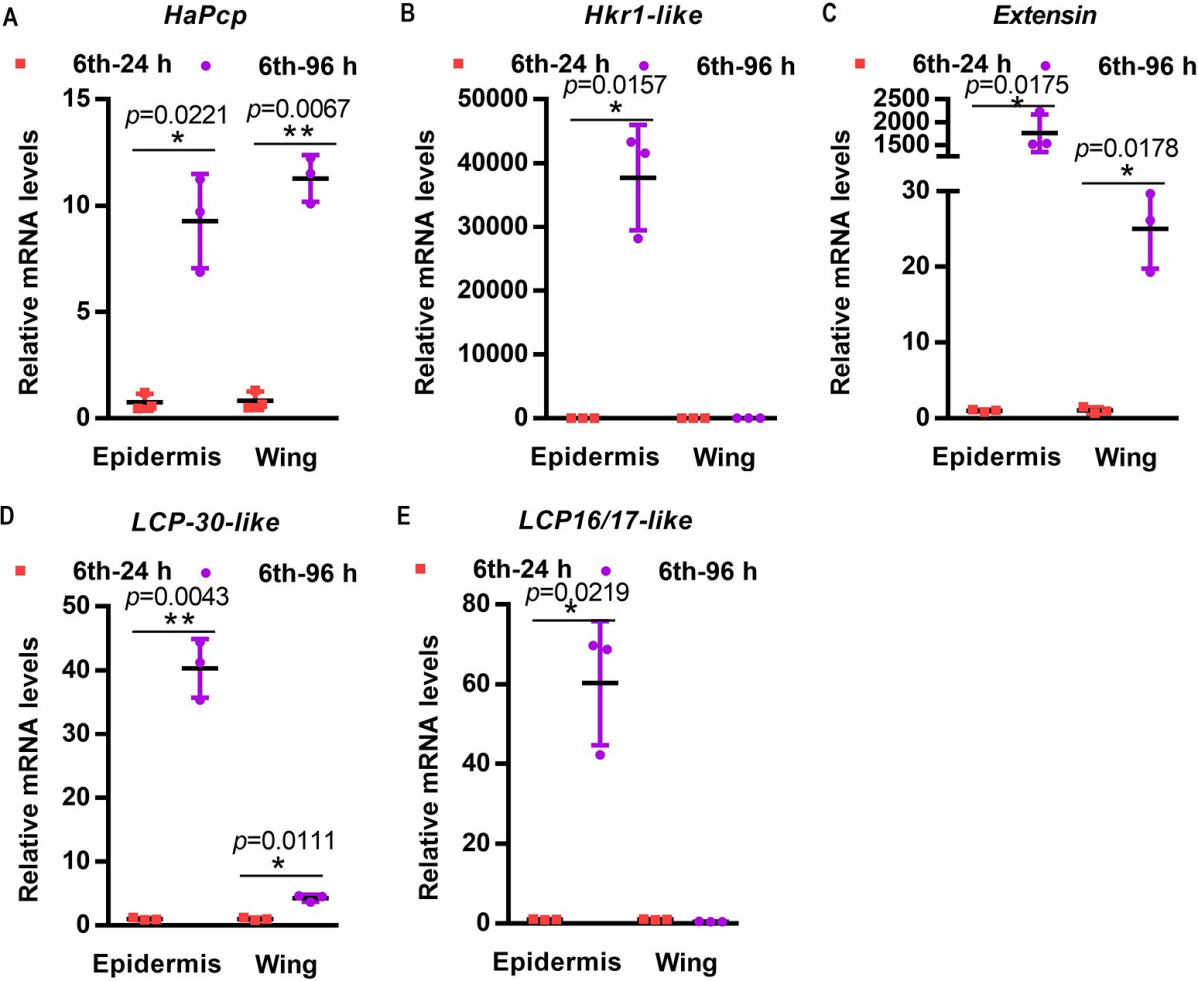
qRT-PCR examining the expression of cuticle proteins. The expression of *HaPcp* (A), *Hkr1-like* (B), *Extensin* (C), *Lcp-30-like* (D), and *Lcp16/17-like* (E) in the epidermis and wings at 6th-24 h and 6th-96 h analyzed by qRT-PCR. All qRT-PCR experiments included three biological replicates and three technical replicates. The error bars represent the mean ± SD, and Student’s *t* test was used to analyze significant differences. *p< 0.05, **p<0.01.

The top five cuticle proteins with the top upregulated expression in the 6th-96 h epidermis and FPKM values greater than 40 in the 6th-72 h wing were selected for qRT-PCR validation. The results confirmed that *HaPcp* and *Extensin* were highly expressed in both the 6th-96 h epidermis and the 6th-72 h wing. *Extensin* is an uncharacterized protein; thus, *HaPcp* was chosen for further study (Fig 2).

### Knockdown of *HaPcp* caused abnormal pupal cuticle and winglessness

**Fig 3:**
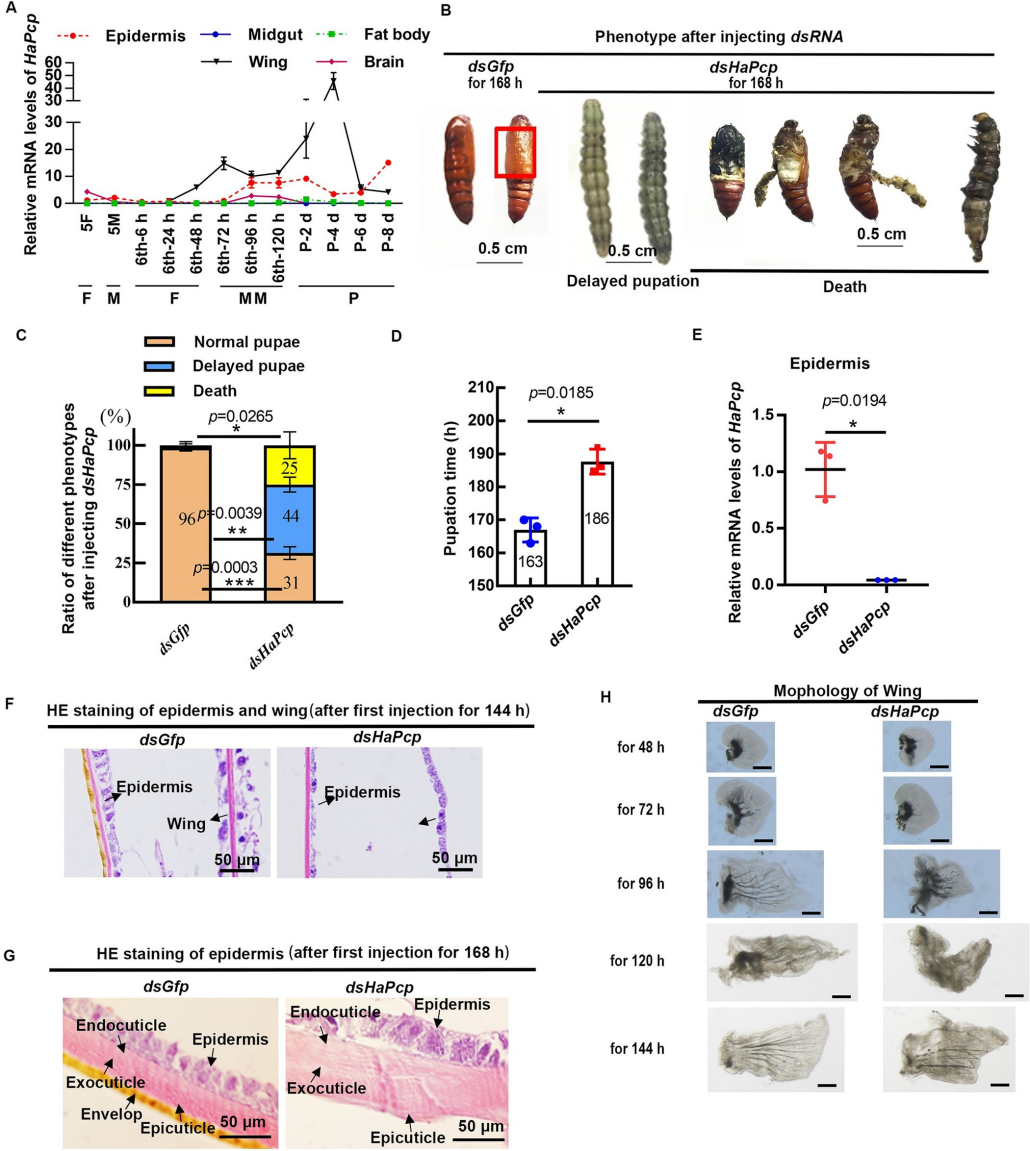
Knockdown of *HaPcp* caused abnormal pupal cuticles and wings. (A) The expression pattern of *HaPcp* was analyzed by qRT-PCR. F: feeding; M: larval molting; MM: metamorphic molting; P: pupae. (B) Morphological differences 168 h after injection of *dsHaPcp* and *dsGfp*. The red box indicates the location of the wings. Ruler 0.5 cm. (C) Statistical analysis of normal pupae, dead pupae, and delayed pupation in the *dsHaPcp* and *dsGfp* groups. Thirty larvae were used for each repetition. (D) Difference in larval pupation time after injection of *dsHaPcp* and *dsGfp*. (E) Epidermal RNA was extracted from 6th-96 h to detect the interference efficiency of *dsHaPcp*. (F) HE staining of epidermis and wings 144 h after the first injection of *dsRNA*. (G) HE staining of epidermis 168 h after the first injection of *dsRNA*. The bars represent 50 μm. (H) Wing morphology in different periods of the *dsGfp* group and *dsHaPcp* group. The bars represent 100 μm. All qRT-PCR experiments included three biological replicates and three technical replicates, error bars represent the mean ± SD, and Student’s *t* test was used to analyze significant differences. *p< 0.05, **p<0.01, ***p<0.001.

To determine when and where *HaPcp* played a role, we performed a qRT-PCR experiment to explore the expression pattern of *HaPcp* at the transcriptional level at different developmental stages. The results showed that the expression of *HaPcp* was high in the epidermis and wings during metamorphosis and was lower in the midgut, fat body and brain during both feeding and metamorphic stages (Fig 3A). This finding suggested that HaPCP plays a major role in the epidermis and wings during metamorphosis.

To examine the role of *HaPcp in vivo*, we carried out RNA interference (RNAi) in larvae. Knockdown of *HaPcp* caused delayed pupation and death with abnormal white abdomen cuticles, winglessness and head abnormalities, and death within one to two days (Fig 3B). Twenty-five percent of the larvae in the *dsHaPcp*-injected group died, and 44% of the larvae exhibited delayed pupation compared with those of the *dsGfp*-injected control group (Fig 3C). The delayed pupation time was 186 h, approximately 23 h later than the average pupation time (163 h) of the control group (Fig 3D). The interference efficiency test showed *HaPcp* interference (Fig 3E). Hematoxylin-eosin staining (HE) staining showed that the abdomen yellow envelope of the pupal cuticle was missing, and the wing had only single layer cells in the *dsHaPcp* group compared to the double layer cells in the control (Fig 3F and 3G). In addition, the wings in the *dsHaPcp* group developed slowly and could not develop into normal wings (Fig 3H). These results showed that HaPCP plays a critical role in cuticle formation and wing development during metamorphosis.

### Transcription factors were highly expressed in the epidermis during metamorphosis

To explore the regulatory mechanism of *HaPcp* expression in the epidermis and wings during metamorphosis, we identified all transcription factors in the epidermis and wings by the transcriptome (S3 Table). Changes in the expression of transcription factors in the 6th-24 h and 6th-96 h epidermis and 6th-72 h wing were analyzed by a Hemi heatmap. Many transcription factors had increased expression with FPKM values >40 in the 6th-96 h epidermis, including hormone receptor HR3 (*Hr3*), *Ovo-like*, *LOC110380141* (unknown protein), *MafK*, WASH complex subunit 3 (*Washc3*), *Pou*, *Runt-like*, *LOC110382070* (unknown), *Sox-12-like*, and NFX1-type zinc finger-containing protein (*Nfx1*). Two transcription factors (*Ovo-like* and *Washc3*) had FPKM values greater than 40 in wings (S4 Fig).

**Fig 4:**
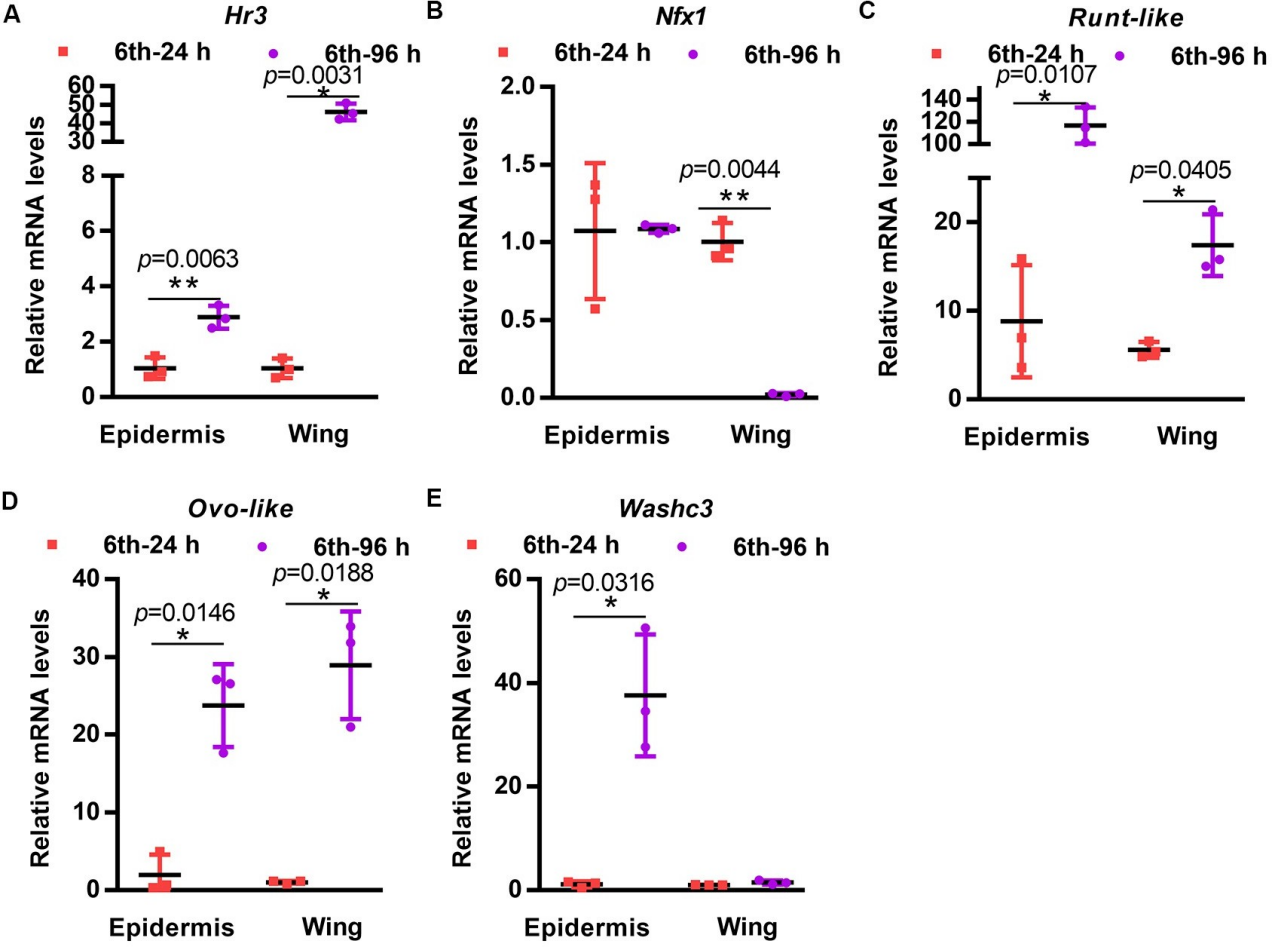
qRT-PCR examining the expression of transcription factors at 6th-24 h and 6th-96 h in the epidermis and wing. (A) The expression of *Hr3*. (B) The expression of *Nfx1*. (C) The expression of *Runt-like*. (D) The expression of *Ovo-like*. (E) The expression of *Washc3*. 6th-24 h: the larvae at the six instar 6 h. 6th-96 h: the larvae at the six instar 96 h. All qRT-PCR experiments included three biological replicates and three technical replicates, error bars represent the mean ± SD, and Student’s *t* test was used to analyze significant differences. *p< 0.05, **p<0.01.

The top five transcription factors (*Hr3*, *Nfx1*, *Runt-like*, *Ovo-like* and *Washc3*) were selected to perform qRT-PCR experiments to verify the transcriptome results. The transcription factor *Hr3* was highly expressed in the wing at 6-96 h; *Nfx1* was not highly expressed in the tissues; *Washc3* was highly expressed in the epidermis at 6-96 h. Only *Runt-like* and *Ovo-like* were expressed in both the 6th-96 h epidermis and the 6th-72 h wings (Fig 4). qRT-PCR partially confirmed the transcriptome results. According to qRT-PCR, *Runt-like* and *Ovo-like*, which had increased expression in both the epidermis and wings, were selected for further study.

### Knockdown of *Runt-like* caused abnormal pupal cuticle and winglessness

**Fig 5:**
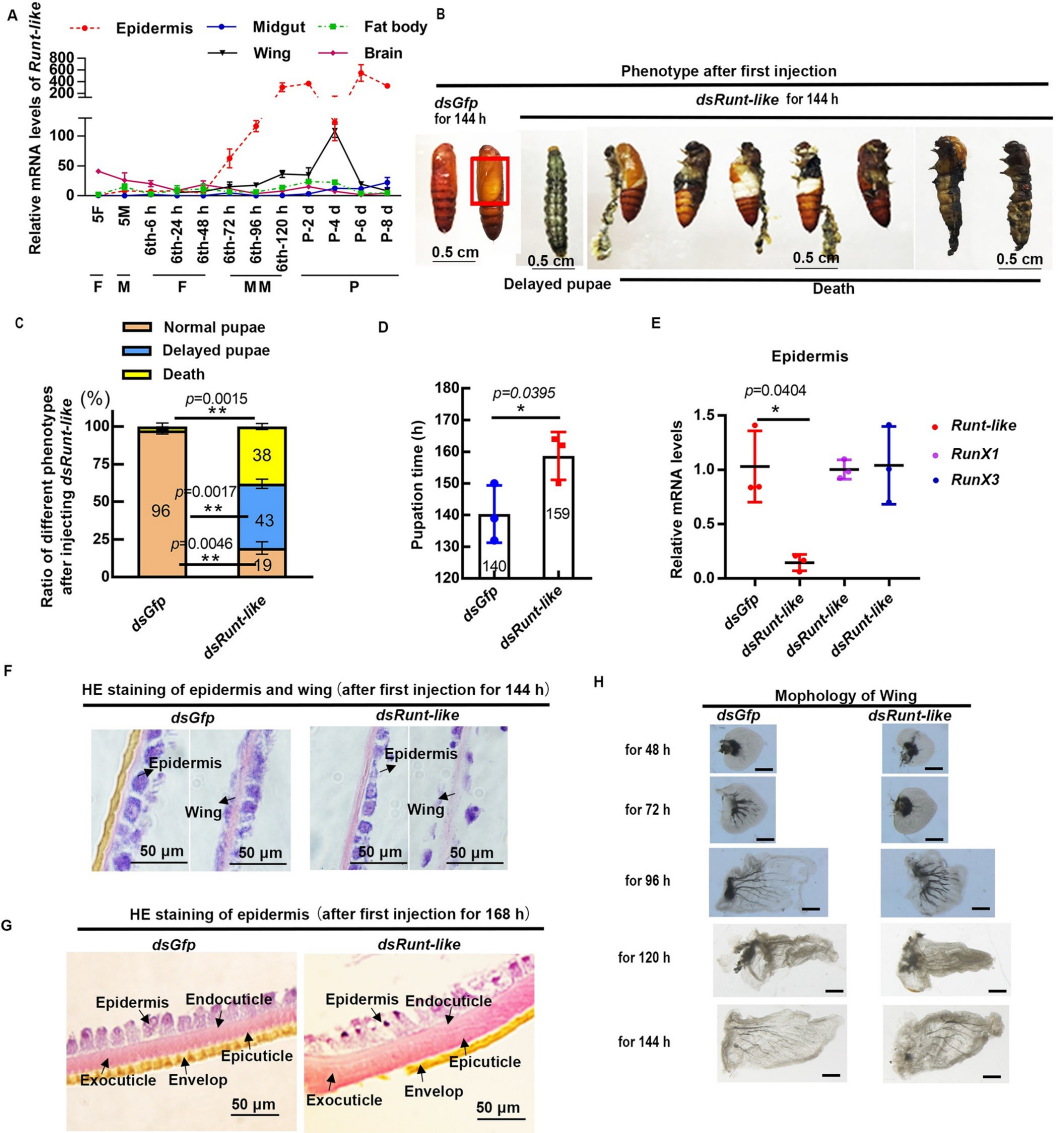
Knockdown of *Runt-like* caused abnormal pupal cuticles and wings. (A) The expression profile of *Runt-like* was analyzed by qRT-PCR. F: feeding; M: larval molting; MM: metamorphic molting; P: pupae. (B) Morphological differences 144 h after injection of *dsRunt-like* and *dsGfp*. Ruler 0.5 cm. (C) Statistical analysis of normal pupae, abnormal and dead pupae, and delayed pupation in the *dsRunt-like* group and *dsGfp* group. Thirty larvae were used for each repetition. (D) Difference in larval pupation time after injection of *dsRunt-like* and *dsGfp*. All qRT-PCR experiments included three biological replicates and three technical replicates, error bars represent the mean ± SD, and a *t* test was used to analyze significant differences. *p< 0.05, **p<0.*01*. (E) Epidermal RNA was extracted from 6th-96 h to detect the interference efficiency of *Runt-like*. *Runx1* and *Runx3* are *Runx* genes, which were examined as the off-target control of *Runt-like* RNAi. (F) and (G) HE staining wing and epidermis after injection of *dsRNA*, respectively. The yellow envelope nearby the absent envelop in HE staining was shown as a control. Ruler 50 μm. (H) Size of the wing. The bars represent 100 μm.

To explore the roles of RUNT-like and OVO-like *in vivo*, we determined the expression profiles. The expression level of *Runt-like* was high in the epidermis during metamorphosis. In addition, *Runt-like* was high in wings at pupal stages compared with in the midgut and fat body (Fig 5A). The expression pattern of *Runt-like* transcript coincides with the changes in the hemolymph titers of the molting hormone 20E. RNAi in larvae showed that the *dsRunt-like* injection group had abnormal cuticles and winglessness, including white color in the anterior abdominal epidermis, wing absence, and an abnormal head, and died within one to two days (Fig 5B). Compared with those of the *dsGfp*-injected control group, 38% of the larvae died in the *dsRunt-like*-injected group, and 43% of the larvae exhibited a delayed pupation phenotype from 140 to 159 h (Fig 5C), which was approximately 19 h later than the average pupation time of the control group (Fig 5D). The interference efficiency test showed that the *Runt-like* gene had been interfered with, and no off-target effects were observed (Fig 5E). HE staining showed that the yellow envelope of the cuticle was absent in the *dsRunt-like* group, and wing cells appeared muddled (Fig 5F and 5G). In addition, the wings of the *dsHaPcp* group developed slowly and could not develop into normal wings (Fig 5H). These results showed a similar phenotype as that in *HaPcp* knockdown, suggesting that RUNT-like plays an important role in cuticle remodeling and wing formation.

After knockdown of *Ovo-like*, the larvae showed delayed pupation and death at larval stages. Forty-six percent of larvae in the *dsOvo-like*-injected group showed a delayed pupation phenotype compared to those in the *dsGfp*-injected control group, and 14% of the larvae showed a death phenotype; however, the phenotype after RNAi of *Ovo-like* did not appear like the *HaPcp* RNAi, in addition to the delayed pupation (S5 Fig), therefore, we did not study *Ovo-like* deeply.

### RUNT-like promoted the transcription of *HaPcp*

**Fig 6:**
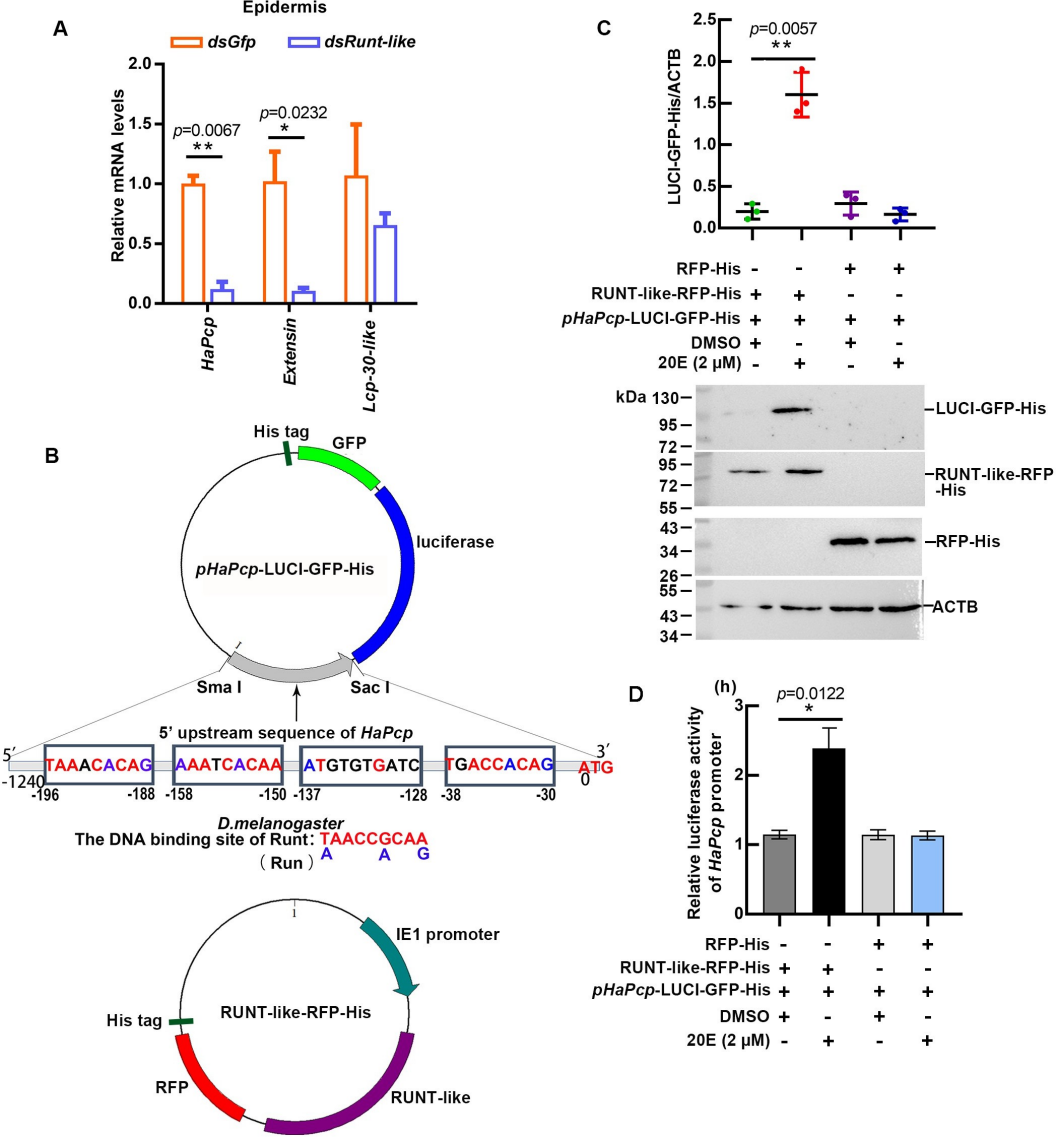
Luciferase assays showing RUNT-like promoting the transcription of *HaPcp*. (A) The expression levels of *HaPcp*, *Extensin*, and *Lcp-30-like* were detected by qRT-PCR after injection of *dsRunt-like*.All qRT-PCR experiments included three biological replicates and three technical replicates, error bars represent the mean ± SD, and a *t* test was used to analyze significant differences (*p<0.05). (B) Plasmid maps of *pHaPcp* -LUCI-GFP-His and RUNT-like-RFP-His, which were overexpressed in HaEpi cells. (C) Western blot detection of LUCI-GFP-His expression. The statistical analysis was by ImageJ. (D) Luciferase assay using a Firefly & Renilla Dual Luciferase Assay Kit. Transcriptional activity of *HaPcp* promoter was detected by dual-luciferase reporter assay. The ordinate represented Fluc/Rluc. Fluc: firefly luciferase; Rluc: Renilla luciferase.

To find the cuticle protein that RUNT-like regulated, we used qRT-PCR to detect the expression changes in the transcript levels of the screened cuticle proteins (*HaPcp*, *Extensin* and *Lcp-30-like*). The results showed that the expression of *HaPcp* and *Extensin* significantly decreased after interference with *Runt-like* in larvae (Fig 6A). Through the online prediction website https://jaspar.genereg.net/, the 5′-upstream regions of these cuticle proteins were shown to contain RUNT binding sites (RUNTbs), and the sequence was highly similar to the binding site T/AAACCG/ACAA/G of *D*. *melanogaster* RUNT and RUN. The 5′-upstream (-1240 nt~-30 nt before ATG) region of *HaPcp* contained 4 RUNT binding sites (RUNTbs) and was constructed as a *pHaPcp*-luciferase-GFP-His reporter plasmid. The full-length sequence of the ORF of RUNT-like was inserted into the pIEx-4-RFP-His plasmid to overexpress RUNT-like-RFP-His. *pHaPcp*-LUCI-GFP-His and RUNT-like-RFP-His plasmids were cotransfected into HaEpi cells, a *H*. *armigera* epidermal cell line (Fig 6B). Western blotting was performed to detect the expression of LUCI-GFP-His when RUNT-like-RFP-His was overexpressed under 20E stimulation but not from the RFP-His control plasmid (Fig 6C), indicated 20E via RUNT-like-RFP-His upregulates *HaPcp* transcription. The luciferase activity assay detected a significant increase in luciferase activity in RUNT-like-RFP-His-overexpressing cells under 20E stimulation (Fig 6D). ChIP results confirmed that RUNT-like-RFP-His enriched more DNA fragments that contain RUNTbs motifs (S6 Fig). These results indicate that 20E via RUNT-like upregulates *HaPcp* transcription.

### 20E upregulated the transcription of *Runt-like* through EcR and FOXO

**Fig 7:**
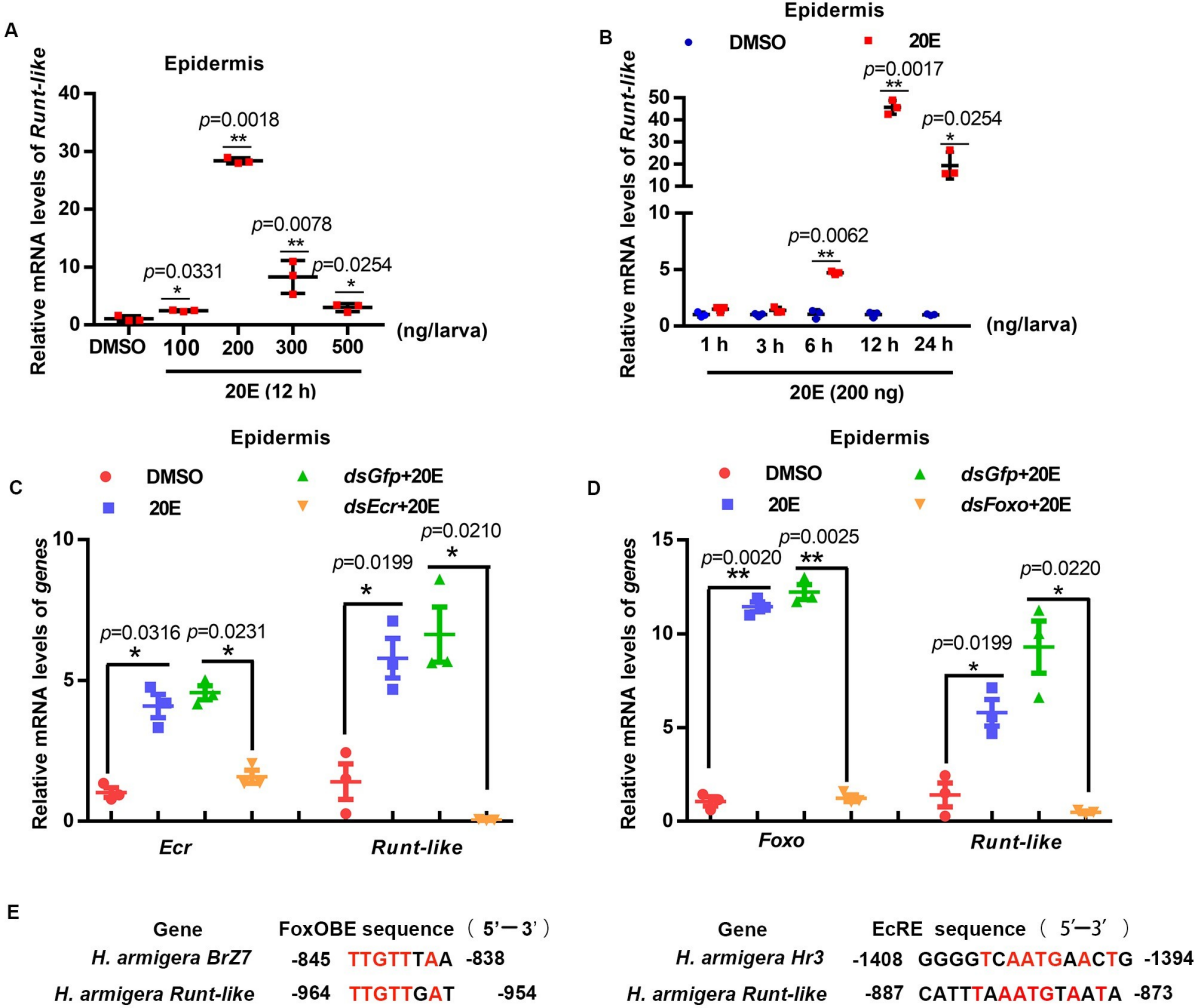
20E regulation of *Runt-like* expression in larvae. (A) The expression of *Runt-like* in the epidermis was measured by qRT-PCR after injecting different concentrations of 20E into the 6th-6 h larvae for 12 h, and the control group was injected with the same amount of DMSO. (B) The 6th-6 h larvae were injected with 200 ng/larva of 20E for different times, and the control group was injected with the same amount of DMSO. Expression of *Runt-like* in epidermis measured by qRT-PCR. (C, D) qRT-PCR analysis of the transcript levels of *Runt-like* after knocking down *Ecr* and *Foxo*. (E) Alignment of FOXOBE sequences between *BrZ7* and *Runt-like* promoters in *H*. *armigera*. Alignment of EcRE sequences in *Hr3* and *Runt-like* promoter regions of *H*. *armigera*. Error bars represent the mean ± SD, and a *t* test was used to analyze significant differences (**p<0.01, *p<0.05).

20E regulation of *Runt-like* expression was examined to confirm the above hypothesis. The results showed that in the epidermis, 20E upregulated the expression of *Runt-like* in a dose-dependent manner (Fig 7A) and a time-dependent manner (Fig 7B). To explore how 20E upregulates the expression of *Runt-like*, we knocked down the nuclear receptor *Ecr* of 20E and the transcription factor *Foxo* by RNAi in larvae. The expression of *Runt-like* was downregulated after interfering with *Ecr* and *Foxo* (Fig 7C and 7D), suggesting that 20E upregulates the expression of *Runt-like* through EcR and FOXO.

The *Runt-like* promoter region was predicted to contain a FOXO binding site (FOXOBE) and EcR binding site (EcRE) by an online prediction website. FOXOBE was predicted as 5’-TTGTTGAT-3’ in the *Runt-like* promoter region, which was highly conserved with the 5’-TTGTTTAA-3’ site of *H*. *armigera BrZ7* [24]. However, the EcRE 5’-CATTTAAATGTAATA-3’ was not conserved with 5’-GGGGTCAATGAACTG-3’ of *Hr3* in *H*. *armigera* [25] (Fig 7E); thus, 20E might upregulate the expression of *Runt-like* through FOXO binding to FOXOBE.

**Fig 8:**
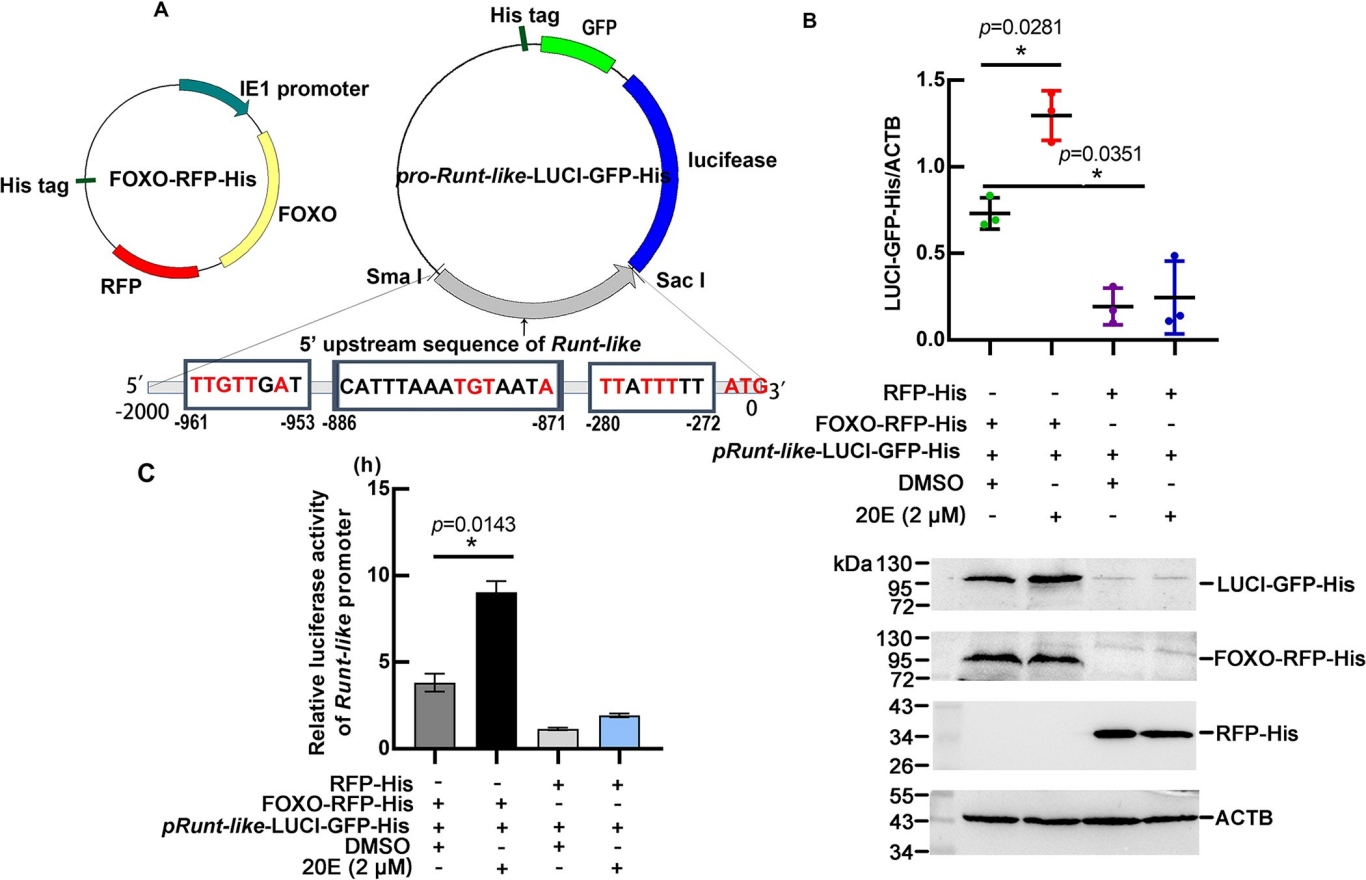
Luciferase assays showing FOXO increasing the transcription of *Runt-like*. (A) Plasmid maps of FOXO-RFP-His and *pRunt-like*-LUCI-GFP-His, which were overexpressed in HaEpi cells. (B) Western blot detection of LUCI-GFP-His expression in HaEpi cells after overexpression of FOXO-RFP-His. Statistical analysis was performed by ImageJ and a *t* test. (C) Luciferase assay using a Firefly & Renilla Dual Luciferase Assay Kit. DMSO was a solvent control for 20E. Error bars represent the mean ± SD, and a *t* test was used to analyze significant differences (*p<0.05).

To confirm this hypothesis, we constructed a *pRunt-like*-LUCI-GFP-His luciferase reporter plasmid containing FOXO binding sites in front of *Runt-like*, and *pRunt-like*-LUCI-GFP-His was cotransfected with the FOXO-RFP-His overexpression plasmid in HaEpi (Fig 8A). Western blotting experiments showed that FOXO-RFP-His could significantly upregulate the expression of LUCI-GFP-His, and the expression was increased by 20E treatment (Fig 8B). A luciferase activity assay showed that FOXO-RFP-His could significantly increase the transcriptional activity of *pRunt-like*-LUCI-GFP-His luciferase (Fig 8C). The ChIP results confirmed that FOXO-RFP-His enriched more DNA fragments that contain the FOXOBE motif (S7 Fig). These results suggested that 20E regulates *Runt-like* transcription via FOXO.

### RUNT-like proteins evolved by increasing the RUNX domain from low to high animals

The phylogenetics of RUNT-like were analyzed to determine its evolution in animals. RUNT proteins, including RUNX, were found from animals, *H*. *sapiens*, *Mus musculus*, *Gallus gallus*, *Xenopus laevis*, *Danio rerio*, *Petromyzon marinus*, *Branchiostoma belcheri*, *Strongylocentrotus purpuratus*, *D*. *melanogaster*, *H*. *armigera*, *Octopus sinensis*, *Caenorhabditis elegans*, *Schmidtea mediterranea*, *Hydra vulgaris*, *Amphimedon queenslandica* and *Tetrahymena thermophile* on the NCBI website and analyzed using Mega 7.0 software to generate a phylogenetic tree. The RUNT-like of *H*. *armigera* was grouped together with *D*. *melanogaster* RUNT-like, separated from the RUNX group (S8 Fig). The protein domain analysis showed that the RUNT domain is conserved in RUNT and RUNX in the evolution of animals; however, a RUNX domain was increased in RUNX proteins (S9 Fig). These results revealed the evolution of RUNT domain-containing proteins.

## Discussion

This work revealed that the cuticle was remodeled from the larval to pupal stage by transcriptional regulation of a set of pupal cuticle protein expression. HaPCP is an important protein for the morphological formation of the pupal cuticle and wing. The transcription factor RUNT-like plays key roles in regulating the transcription of the pupal cuticle protein HaPCP, and 20E, via FOXO binding to the promoter of *Runt-like*, directly regulates *Runt-like* transcription.

### HaPCP participates in pupal cuticle development

The larval cuticle and the pupal cuticle are very different in morphology, so the replacement of the larval cuticle by the pupal cuticle and the larval epidermal proteins by the pupal epidermal proteins must be performed during the transition from larva to pupa. Cuticle proteins are also the main component of insect wings and play an important role in the development of wings. WCPs are a class of wing disc development proteins that are specifically expressed in the epidermis and are responsible for the formation of the pupal epidermis during the transition from larvae to pupae. This study revealed a new pupal cuticle protein, HaPCP, which is related to pupal cuticle and wing development. However, the role of HaPCP in wing development needs further study, in addition to the absent of the envelop and abnormal cell layers of wing.

The nomenclature of insect cuticle is reinforced as an external compartment made from three layers, including envelope, epicuticle on the inner face of the envelope, and the procuticle assembles at the cell surface [2]. The cuticle proteins have conserved Rebers-Riddiford motif (RR), a chitin bind motif, which is different from the cysteine-containing chitin-binding domain found in chitinases and some peritrophic membrane proteins. The cuticle protein is confirmed binding chitin in mosquito *Anopheles gambiae* [26]. The cuticle protein RR-1 of *B*. *mori* is a chitin binding protein mainly found in cuticle [27]. HaPCP is a cuticle protein like the cuticle protein RR-1 of *B*. *mori*, however, we found the knockdown of *HaPcp* resulted in the lack of abdomen yellow envelope. This result rises an interesting question that how a cuticle protein affects envelope, because the envelope is mainly consisted of lipids and quinones [3]. One possibility is HaPCP affects envelope formation by constructing the structure of cuticle and materials transport. The *Tribolium castaneum* RR-1 cuticular protein TcCPR4 is required for the formation of pore canals in rigid cuticle [28]. The cuticular protein 49Ah in *D*. *melanogaster* is involved in a nanoscale protrusion formation on corneal lens cell polarity/cell architecture in eye development [29]. In addition to the development of the envelop, our study also observed abnormal wing and head after knockdown of *HaPcp*, suggesting HaPCP plays other roles in the development, which need to be addressed in future study.

### RUNT-like regulates *HaPcp* transcription

*DmRunt* (AAC27780.1) is a pair-rule gene and is part of the network of genes that control pattern formation in the embryo. In the central nervous system, RUNT function is necessary for the development of a subset of neurons. RUNT is also a key regulator of sex determination and directly controls sex-lethal, a master gene that determines the sex of the animal and controls dosage compensation [18]. In *D*. *melanogaster*, RUNT and Lozenge (another RUNX family protein) (NP 511099.2) regulate various developmental processes [23]. DmRUNT in *D*. *melanogaster* (AAC27780.1) plays a key role in embryonic neural development [17, 23]. The RUNT-like protein in this study has the highest homology with DmRUNT. *H*. *armigera Runt-like* was highly expressed in the epidermis and wings during metamorphosis to play a role in promoting the formation of the pupal cuticle by upregulating *HaPcp* transcription in *H*. *armigera*. However, the mechanism of RUNT-like in wing development needs further study.

### 20E via FOXO regulates *Runt-like* transcription

Previous studies have found that the expression of insect cuticle proteins is stage-specific and regulated by 20E, and some cuticle protein genes are specifically expressed during metamorphosis [30], including the cuticle protein genes *Edg84a * and *Edg78e* of *D*. *melanogaster* pupa [31] and the cuticle protein genes *Bmwcp10*, *Bmwcp2* and *Bmwcp5* of *B*. *mori* pupa [32]. The transcription factors βFTZ-F1, BR-C Z2 or POUM2 of 20E pathway will combine with the promoter region of the pupal cuticle protein-encoding gene to promote the expression of the pupal cuticle protein-encoding gene [33]. In *B*. *mori*, the transcription factor BmβFTZ-F1 binds to the upstream promoter region of the wing disc cuticle protein-encoding gene *BmWCP5* of *B*. *mori* to promote the expression of the cuticle protein and then participates in the formation of wing discs during the transformation of larvae to pupae [6]. Here, we revealed that RUNT-like plays an important role in upregulating the cuticle protein *HaPcp* in pupal cuticle formation and wing development, whereas FOXO directly upregulates *Runt-like* transcription by binding to FOXOBE. FOXO is already known to be upregulated by 20E via its nuclear receptor EcR [12]; thus, 20E upregulated *Runt-like* transcription via EcR and FOXO.

There are many cuticle proteins and transcription factors differentially expressed during larval-pupal metamorphosis in the epidermis and wing, however we have only selected top 5 candidates each for the study. There must be other genes involved in pupal cuticle development, which need to be study in future work. In addition, 20E regulates gene expression by regulating the post-translational modification of the transcription factor. For examples, FOXO is non-phosphorylated thus translocated into nucleus to promote gene transcription under 20E regulation [12]. EcRB1 is phosphorylated and interacts with USP to form transcription complex to promote gene transcription [34]. The different responses of the overexpressed RUNT-like-RFP-His and FOXO-RFP-His to 20E in HaEpi cells suggests RUNT-like-RFP-His and FOXO-RFP-His need to be regulated both at transcriptional levels and at the post-transcriptional levels for protein interaction by 20E.

## Conclusion

**Fig 9:**
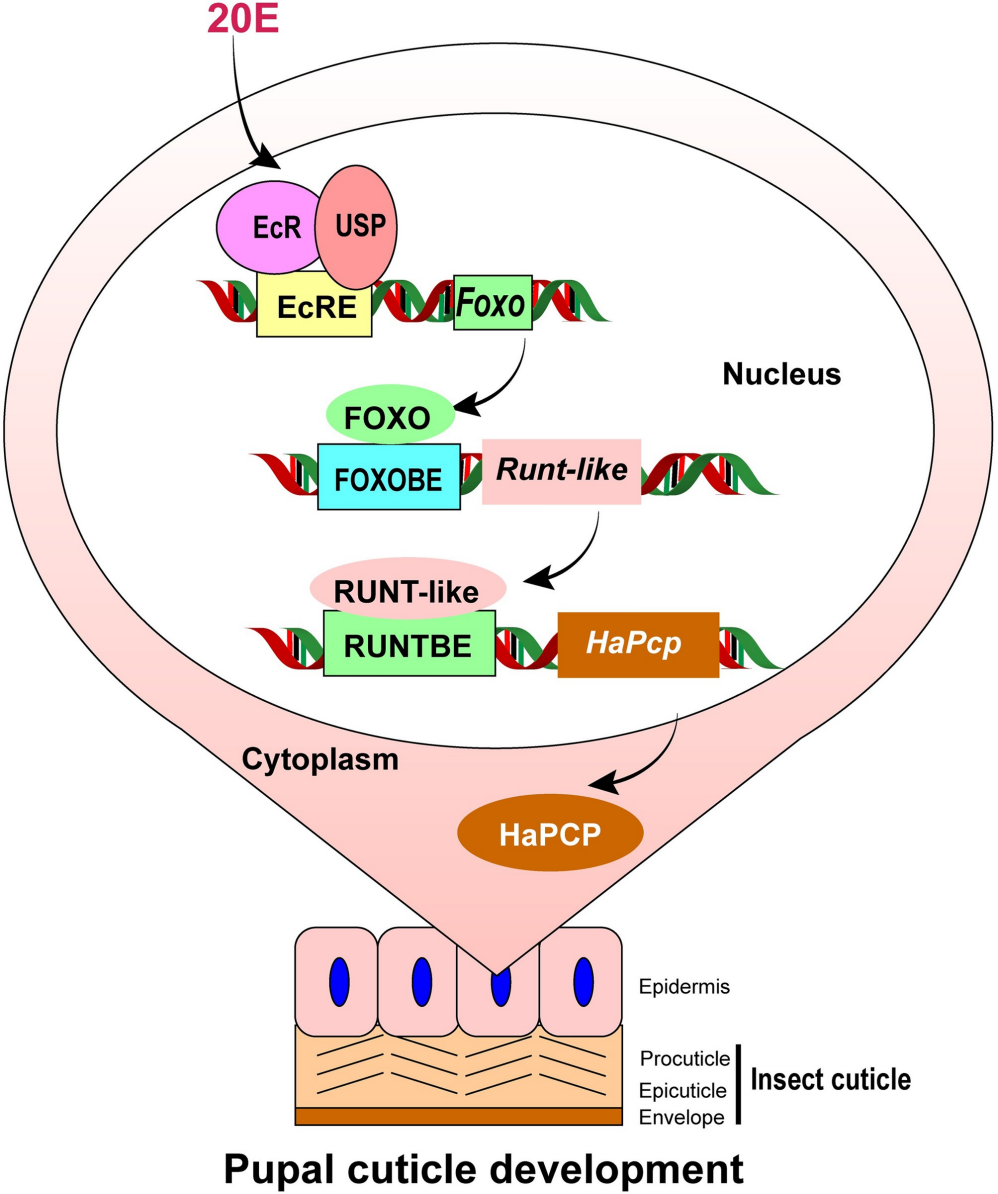
Chart to illustrate the roles and regulation of RUNT-like and HaPCP in pupal cuticle development. 20E promotes the expression of RUNT-like through FOXO, thus promoting the expression of the pupal cuticle protein HaPCP during metamorphosis to form the envelop of pupal cuticle. The nomenclature of insect cuticle is according to reference [2], in which, procuticle includes endocuticle and exocuticle.

20E promotes the high expression of *Runt-like* in the epidermis and wing through FOXO during metamorphosis. Then, RUNT-like promotes the transcription of cuticle protein *HaPcp*. High expression of HaPCP contributes envelope formation in pupal cuticle and wing development during metamorphosis (Fig 9).

## Experimental materials and methods

### Experimental animals

The animals used in the experiment were *H*. *armigera* purchased from Taobao (https://m.tb.cn/h.fndG4sk?tk=NtaE2VawFQy). They were fed artificial feed. The living environment temperature was 25°C-27°C, the humidity was 40% - 60%, and the daily light duration was 14 h.

### Transcriptome sequencing

The samples were collected from the epidermis of the 6th instar larvae at 24 h (6th-24 h Epi), the epidermis of the 6th instar larvae at 96 h (6th-96 h Epi), and the wing disc in the 6th instar larvae at 72 h (6th-72 h wing). The second-generation parametric transcriptome sequencing was performed by Illumina sequencing plate in Gooal gene company (www. Gooalgene.com, in Wuhan, China). The RNA was extracted and the libraries were constructed. The sequencing produced 6 Gb data in the well controlled quality of the libraries.

### Transcriptome data analysis

We sequenced the 6th-24 h larval epidermis and 6th-96 h larval epidermis from multiple larvae as a sample and identified all cuticle proteins and transcription factors in the transcriptome (S1 and S2 Tables). Hemi software was used to make a heatmap to analyze whether these genes were stage-specifically expressed. Then, these cuticle proteins and transcription factors were classified according to the FPKM value; then, the differentially expressed Cps and transcription factors with log (6th-24 h Epi FPKM/6th-96 h Epi FPKM) greater than 2 were screened out; finally, genes with FPKM values greater than 40 were screened out of these differentially expressed epidermal proteins and transcription factors. Among these genes, the first three genes with greater differences were the target genes.

### Bioinformatics analysis

We identified all the RUNXs of *H*. *sapiens*, *D*. *melanogaster* and *Rattus norvegicus* on the National Center for Biotechnology Information (NCBI) website (https://www.ncbi.nlm.nih.gov/) and obtained all the RUNXs in *H*. *armigera* by basic local alignment search (BlAST) in the *H*. *armigera* genome (https://blast.ncbi.nlm.nih.gov/Blast.cgi?PROGRAM=blastp&PAGE_TYPE=BlastSearch&BLAST_SPEC=OGP__29058__158185&LINK_LOC=blasttab&LAST_PAGE=blastp#). Phylogenetic tree analysis was performed using MAGE 7.0. Sequence alignment was performed using DNAMAN software. The molecular weight and isoelectric point of the protein were predicted using the ExPASy Compute pI/Mw website (https://web.expasy.org/compute_pi/).

### Gene cloning

Three larvae in each group were dissected, and 100 mg of each of their epidermis, midgut, fat body and brain were taken, washed, and lysed with TRIzol, and RNA was extracted according to their instructions. Extracted RNA was reverse-transcribed at 5× in 1 RT MasterMix Reverse Transcription Kit (Abm, Zhenjiang, China) to obtain cDNA. With cDNA as a template, specific primers were designed using Primer software, and the gene sequence was amplified by PCR with the primers (S3 Table).

### Quantitative real-time polymerase chain reaction (qRT-PCR)

A 10 μL system was prepared in the dark: 5 μL of 2 × fluorescent PCR Mix, 1 μL of cDNA template, 2 μL of F primer and 2 μL of R primer. The program was set as 95°C for 10 min, 95°C for 10 s, and 60°C for 1 min. qRT-PCR was run using the primers (S3 Table), and data analysis was performed with the given Ct values.

### RNA interference (RNAi)

The control group and the experimental group were selected from 40 *H*. *armigera* at 6th-6 h. The *dsRNA* was diluted to 400 ng/μL with sterilized phosphate-buffered saline (PBS) on a sterile ultraclean bench, and 5 μL of *dsRNA* was injected into each 6th-6 h larva with a microinjector. The larvae were injected once every 24 hours for a total of four injections. Eighteen hours after the fourth injection (6th-96 h), 3 larvae were randomly selected from each group to extract tissue RNA and protein, and qRT-PCR was used to detect the interference efficiency. Changes in epidermal protein-encoding gene expression were observed; embedded sections and hematoxylin-eosin staining (HE) staining were used to observe changes in epidermal morphology and structure; and morphological changes were observed in larval epidermis, pupation and mortality.

### Western blotting

Fifty micrograms and 20 μg of dissected tissue and cell proteins were loaded on a 12.5%-15% SDS-PAGE gel and then transferred to a nitrocellulose membrane, which was washed with 5% nonfat dry milk. The blocking solution prepared with 95% TBS (10 mM Tris-HCl, 150 mM NaCl, pH 7.5) was blocked for 1 h at room temperature, and the primary antibody was diluted with the blocking solution (1:5000 dilution of ACTB rabbit mAb) at 4°C The membrane was incubated overnight, washed twice with TBST (TBS with 0.02% Tween), washed once with TBS, at 10 min/wash, and incubated at room temperature with a 1:5000 dilution of secondary antibody (goat anti-rabbit IgG/alkaline phosphatase labeling) in blocking solution The membrane was incubated at 37°C for 0.5 h, using the same anti-washing method, with TBS chromogenic solution containing 5% NBT and 5% BCIP protected from light.

### Hematoxylin and eosin (HE)

The insect body was placed in PBS, and the tissues were dissected carefully under a microscope. The tissue samples were immersed in 4% paraformaldehyde, shielded from light, and sent to Servicebio (Wuhan, China) for slice and HE staining. After being dewaxed, the slides were stained with hematoxylin for 3–5 min at room temperature, washed one min by water, differentiated by 1% hydrochloric acid alcohol for 20 s, washed i min by water, scott promoted blue for 1 min, and washed i min by water, dewater and stained by eosin dye solution for 5 min, dewater, and sealed and observed under an Olympus BX51.

### Luciferase assays

The 1240 bp promoter region including RUNT binding site sequence of *HaPcp* was cloned into the promoter region of the luciferase-GFP-His plasmid constructed in our laboratory and transfected into HaEpi, along with an internal reference, plasmid phRL-TK. A total of four groups were transfected; the first group was prepared and transfected with Runt-like-RFP overexpression plasmid, and DMSO was added after 6 h as a control; the second group was prepared and transfected with Runt-like-RFP-His overexpression plasmid, and 20E hormone stimulation was performed after 6 h; and the third group was prepared and transfected with RFP overexpression plasmid. For the expression plasmid, DMSO was added as a control after 6 h; the fourth group was transfected with the RFP overexpression plasmid, and 20E hormone stimulation was performed after 6 h. After 72 h, the transcriptional efficacy of the transcription factor was detected with a Firefly & Renilla Dual Luciferase Assay Kit (US Everbright, Inc., F6075).

### 20E stimulation of the larval body

20E was diluted with sterile PBS on a clean bench to a concentration gradient of 20, 40, 60, and 100 ng/μL, 15 *H*. *armigera* were selected at 6-6 h, and 5 μL was injected into 3 larvae for each concentration. The final concentrations were 100, 200, 300, and 500 ng/larva. The control group was injected with the same volume of dimethyl sulfoxide (DMSO into 3 larvae) and stimulated for 12 h. *In vivo*, the larvae were stimulated for 1, 3, 6, 12, and 24 h, and the control group was injected with 5 μL of DMSO into 15 larvae of the same instar stage and stimulated for the same time. Larval epidermal RNA was extracted for subsequent detection.

### Chromatin immunoprecipitation (ChIP)

Five micrograms of pIEX-RFP-His, pIEX-FOXO-RFP-His and pIEX-RUNT-like-RFP-His plasmids was transfected into *H*. *armigera* epidermal (HaEpi) cells, respectively, to culture for 72 h, followed by stimulation with DMSO or 20E for 6 h. The ChIP kit (P2078, Beyotime Biotechnology, Shanghai, China) instructions were followed for the experiment. Then, 40.5 μL of formaldehyde was added to each well and incubated at 37°C in the dark for 10 min. Then, 165 μL of glycin was added and mixed and placed at room temperature for 5 min. After the removal of formaldehyde and glycine, 1 mL of 1×PBS containing PMSF was added to wash the cells twice, and the cells were collected with a cell scraper. The cell precipitate was suspended in SDS buffer containing PMSF, and ultrasonic fragmentation was performed to shear the genomic DNA so that most of the DNA was broken into 200–1000 bp fragments. After centrifugation at low temperature, 400 μL of supernatant was removed from each cell suspension, and 500 μL of dilution buffer was added. The supernatant was collected after centrifugation and evenly divided into three parts, one of which was the input group and placed in the refrigerator at 4°C. The other two parts were added to protein A+G and shaken at 4°C for 30 min to remove specific binding. After centrifugation of the samples at low temperature, the supernatant was taken and divided into two parts, one with 3 μL of antibody and the other without antibody, and the samples were placed in a 4°C shaker overnight. Sixty microliters of protein A+G was added to the samples and shaken for 1 h at 4°C to precipitate the protein recognized by the primary antibody. After the supernatant was removed by centrifugation at low temperature, the precipitate was washed with low salt wash buffer, high salt wash buffer, LiCl wash buffer and TE buffer. Then, 250 μL of elution buffer (1% SDS, 0.1 M NaHCO_3_) was added and eluted in a shaker for 5 min. After this step was repeated, a total of 500 μL of supernatant was collected, and 20 μL of 5 M NaCl was added. After mixing, the solution was heated at 65°C for 4 h. The samples were mixed with 10 μL of 0.5 M EDTA, 20 μL of 1 M Tris (pH 6.5) and 1 μL of 20 mg/mL protease K and incubated at 45°C for 60 min. Finally, chloroform was added to extract DNA for qRT-PCR detection.

### Statistics

The experiments were repeated three times, and the qRT-PCR was repeated three times biologically and three times technically. The significant difference between the two samples was determined by two-tailed paired *t* test. Data were generated using GraphPad 7.0. ANOVA for multiple comparisons.

### Antibody

The antibodies used in the study are commercial products. The primary antibodies of Anti RFP-Tag Mouse Monoclonal Antibody, Anti GFP-Tag Mouse Monoclonal Antibody and ACTB Rabbit mAb were purchased from ABclonal company (Wuhan, China). The secondary antibody is a Goat Anti Rabbit IgG-AP purchased from ZSBio (Beijing, China).

## Supporting information

S1 Fig Phylogenetic tree and domain of cuticle proteins. (A) Phylogenetic analysis of the proteins. (B) Domain analysis of the protein. Red dots indicate *H*. *armigera Rpb1-like* (renamed to *HaPcp* now); blue dots indicate other cuticle proteins in *H*. *armigera*. Triangles indicate DNA-directed RNA polymerase II subunit RPB1 in *H*. *armigera*. Domain of cuticle proteins from different species.
(TIF)

S2 Fig Alignment of the cuticle proteins. The sequences are: *H*. *armigera* RPB1-like (renamed *HaPcp* now in this work), XP_021186897.1; *H*. *armigera* hypothetical proteinB5X24_HaOG214610 (100% identity to RPB1-like, and renamed *HaPcp* now), PZC80453.1; *Bombyx mori* cuticular protein RR-1 motif 21 isoform X1, XP_021205858.1; *Spodoptera litura* cuticular protein RR-1, TKX27920.1; *Drosophila melanogaster*, cuticular protein 49Ah; *Tribolium castaneum* pupal cuticle protein 20, XP_968434.1; *T*. *castaneum* Tc_04500 protein, ACN43338.1. The *B*. *mori* chitinase isoform X1, XP_037867787.1 was used as an indication of other kind of chitin binding protein, which is different to cuticle proteins. The red box indicates chitin binding domain predicted by SMART software (http://smart.embl-heidelberg.de/smart/set_mode.cgi?NORMAL=1).
(TIF)

S3 Fig Prediction of the chitin binding of HaPCP. (A) Whole structure and docking of HaPCP. (B) The magnified docking site. The red stick is the chitin. The magenta dots show the hydrogen bond between chitin and the amino acids: 54N, 61Q, 220E and 224K, which are conserved in *H*. *armegera* and *Heliothis virescens*. The structure of HaPCP was predicted by AlphaFold Protein Structure Database by reference of *Heliothis virescens* (90% identity) (https://alphafold.ebi.ac.uk/). The structure of chitin (C8H13NO5)N was from Pubchem (https://pubchem.ncbi.nlm.nih.gov/substance/162176842). Software SYBYL-X 2.0 was used for docking of the protein and chitin. PyMol was used to view the structure of the protein and chitin.
(TIF)

S4 Fig Expression analysis and screening of transcription factors in the epidermis and wings of *H*. *armigera*. (A) A Hemi heatmap was used to analyze the expression of all transcription factors in the 6th-24 h epidermis, 6th-96 h epidermis and 6th-72 h wings. (B) FPKM value analysis of transcription factors in the 6th-24 h epidermis, 6th-96 h epidermis and 6th-72 h wing. The vertical axis represents the number of genes. (C) Transcription factors with upregulated expression in the 6th-96 h epidermis and FPKM values greater than 40 in the 6th-96 h epidermis and 6th-72 h wings. The vertical axis represents the value of log2 (6th-96 h Epi FPKM/6th-24 h Epi FPKM). The hollow stars represent seven transcription factors that are upregulated in the 6th-96 h epidermis and have FPKM greater than 40. The solid stars represent the transcription factors with FPKM values greater than 40 in the 6th-72 h wing of the ten transcription factors.
(TIF)

S5 Fig Knockdown of *Ovo-like* caused delayed pupation. (A) Morphological differences after 144 h injection of *dsOvo-like* and *dsGfp*. Ruler 0.5 cm. (B) Statistical analysis of normal pupae, dead pupae, and delayed pupation in the *dsOvo-like* group and *dsGfp* group. Thirty larvae were used for each repetition. (C) Difference in larval pupation time after injection of *dsOvo-like* and *dsGfp*. (D) Epidermal RNA was extracted from 6th-96 h to detect the interference efficiency of *dsOvo-like*. All qRT-PCR experiments included three biological replicates and three technical replicates, error bars represent the mean ± SD, and a *t* test was used to analyze significant differences (*p<0.05, **p<0.01).
(TIF)

S6 Fig ChIP experiment showing that 20E promoted *HaPcp* transcription via RUNT-like binding to the RUNTbs in the HaPcp promotor. The primer RUNT of *HaPcp* is the *HaPcp* promoter sequence containing the RUNT-like binding site. The primer *HaPcp*, as a non-RUNT-like binding site control, targets the *HaPcp* open reading frame (ORF). IgG, nonspecific rabbit IgG.
(TIF)

S7 Fig ChIP experiment showing that 20E promoted *Runt-like* transcription via the FOXO binding site in the *Runt-like* promoter region. **A.** ChIP analysis of the FOXO binding site in the *Runt-like* promoter region. FOXO-RFP-His or RFP-His was overexpressed in HaEpi cells for 72 h. IgG, nonspecific rabbit IgG. Primer FOXOBE of *Runt-like*: primer targeted to the *Runt-like* promoter FOXOBE-containing sequence. Primer *Runt-like*: primer targeted to *Runt-like* ORF. **B.** ChIP experiment showing that 20E promoted *Runt-like* expression via FOXO binding to FOXOBE, as measured by qRT-PCR. The FOXOBE primer of *Runt-like* is the *Runt-like* promoter sequence containing FOXOBE. The primer *Runt-like* was used as a non-FOXOBE control targeting the *Runt-like* ORF. IgG, nonspecific rabbit IgG.
(TIF)

S8 Fig Phylogenetic tree analysis of RUNX from different species with the corresponding sequences. (TIF)

S9 Fig Domain analysis of RUNX from different species with the corresponding sequences. (TIF)

S1 Table Cuticle protein. (DOCX)

S2 Table Transcription factors. (DOCX)

S3 Table Primers used in the experiments. (DOCX)

S1 Data The original data that were used to produce the figures in this study. (PDF)
