# Supplementary figures and images for: The transcription factor RUNT-like regulates pupal cuticle development via promoting a pupal cuticle protein transcription
Source: PLoS Genet. 2024 Sep 12;20(9):e1011393. doi: 10.1371/journal.pgen.1011393 (PMC11392391; doi:10.1371/journal.pgen.1011393)

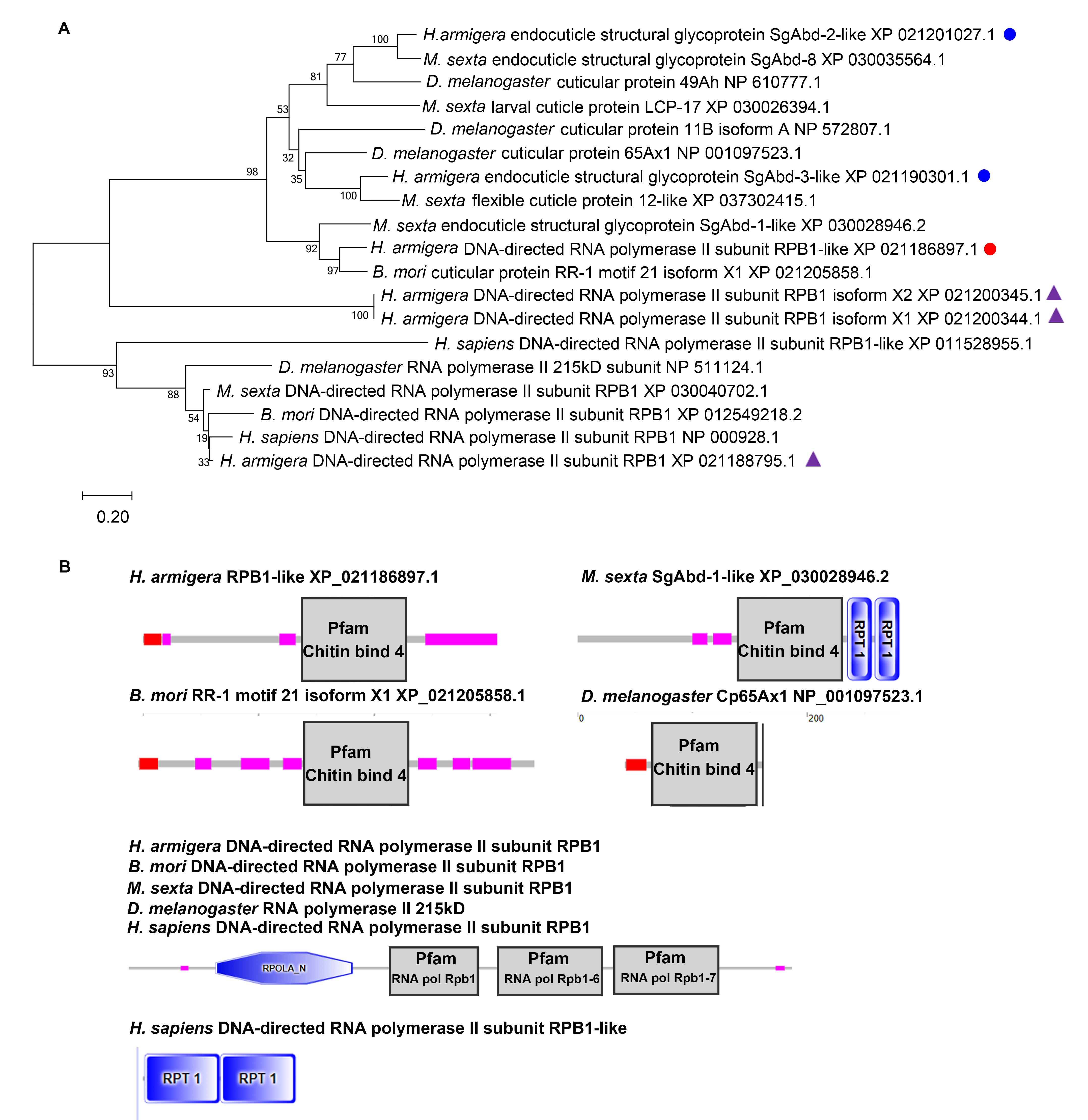

Supplement: S1 Fig — (A) Phylogenetic analysis of the proteins. (B) Domain analysis of the protein. Red dots indicate H. armigera Rpb1-like (renamed to HaPcp now); blue dots indicate other cuticle proteins in H. armigera. Triangles indicate DNA-directed RNA polymerase II subunit RPB1 in H. armigera. Domain of cuticle proteins from different species. (TIF) [file pgen.1011393.s001.tif]

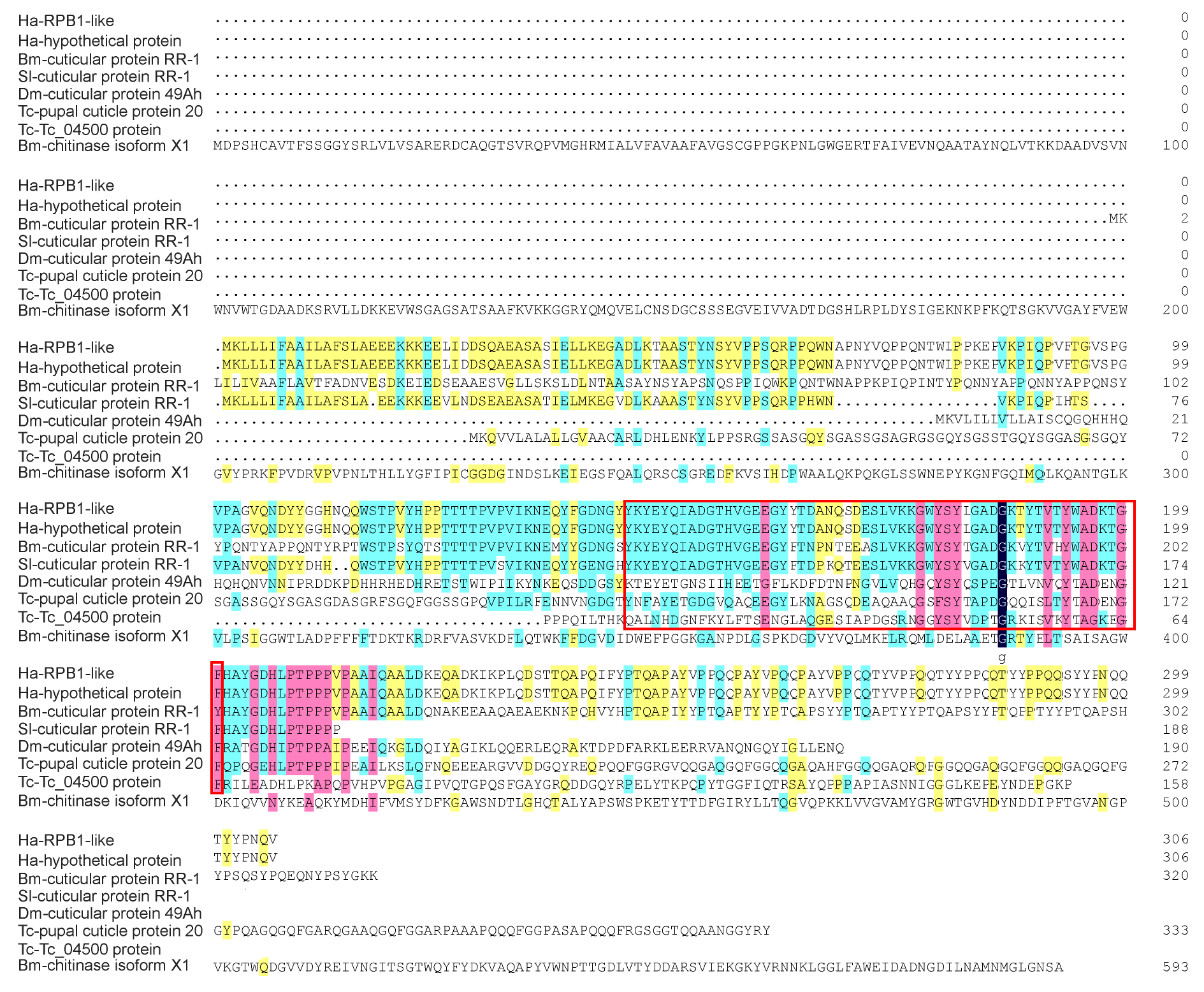

Supplement: S2 Fig — The sequences are: H. armigera RPB1-like (renamed HaPcp now in this work), XP_021186897.1; H. armigera hypothetical proteinB5X24_HaOG214610 (100% identity to RPB1-like, and renamed HaPcp now), PZC80453.1; Bombyx mori cuticular protein RR-1 motif 21 isoform X1, XP_021205858.1; Spodoptera litura cuticular protein RR-1, TKX27920.1; Drosophila melanogaster, cuticular protein 49Ah; Tribolium castaneum pupal cuticle protein 20, XP_968434.1; T. castaneum Tc_04500 protein, ACN43338.1. The B. mori chitinase isoform X1, XP_037867787.1 was used as an indication of other kind of chitin binding protein, which is different to cuticle proteins. The red box indicates chitin binding domain predicted by SMART software (http://smart.embl-heidelberg.de/smart/set_mode.cgi?NORMAL=1). (TIF) [file pgen.1011393.s002.tif]

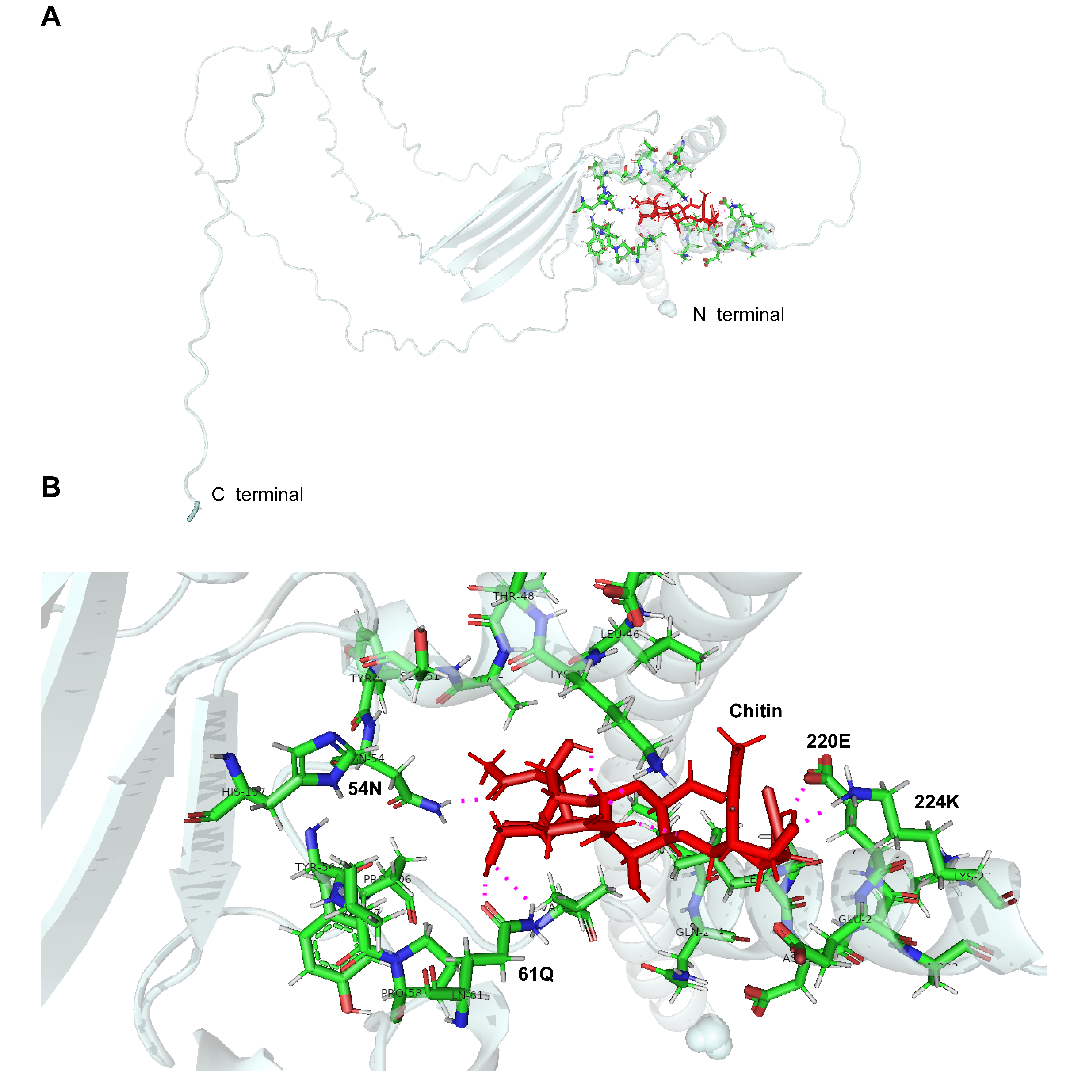

Supplement: S3 Fig — (A) Whole structure and docking of HaPCP. (B) The magnified docking site. The red stick is the chitin. The magenta dots show the hydrogen bond between chitin and the amino acids: 54N, 61Q, 220E and 224K, which are conserved in H. armegera and Heliothis virescens. The structure of HaPCP was predicted by AlphaFold Protein Structure Database by reference of Heliothis virescens (90% identity) (https://alphafold.ebi.ac.uk/). The structure of chitin (C8H13NO5)N was from Pubchem (https://pubchem.ncbi.nlm.nih.gov/substance/162176842). Software SYBYL-X 2.0 was used for docking of the protein and chitin. PyMol was used to view the structure of the protein and chitin. (TIF) [file pgen.1011393.s003.tif]

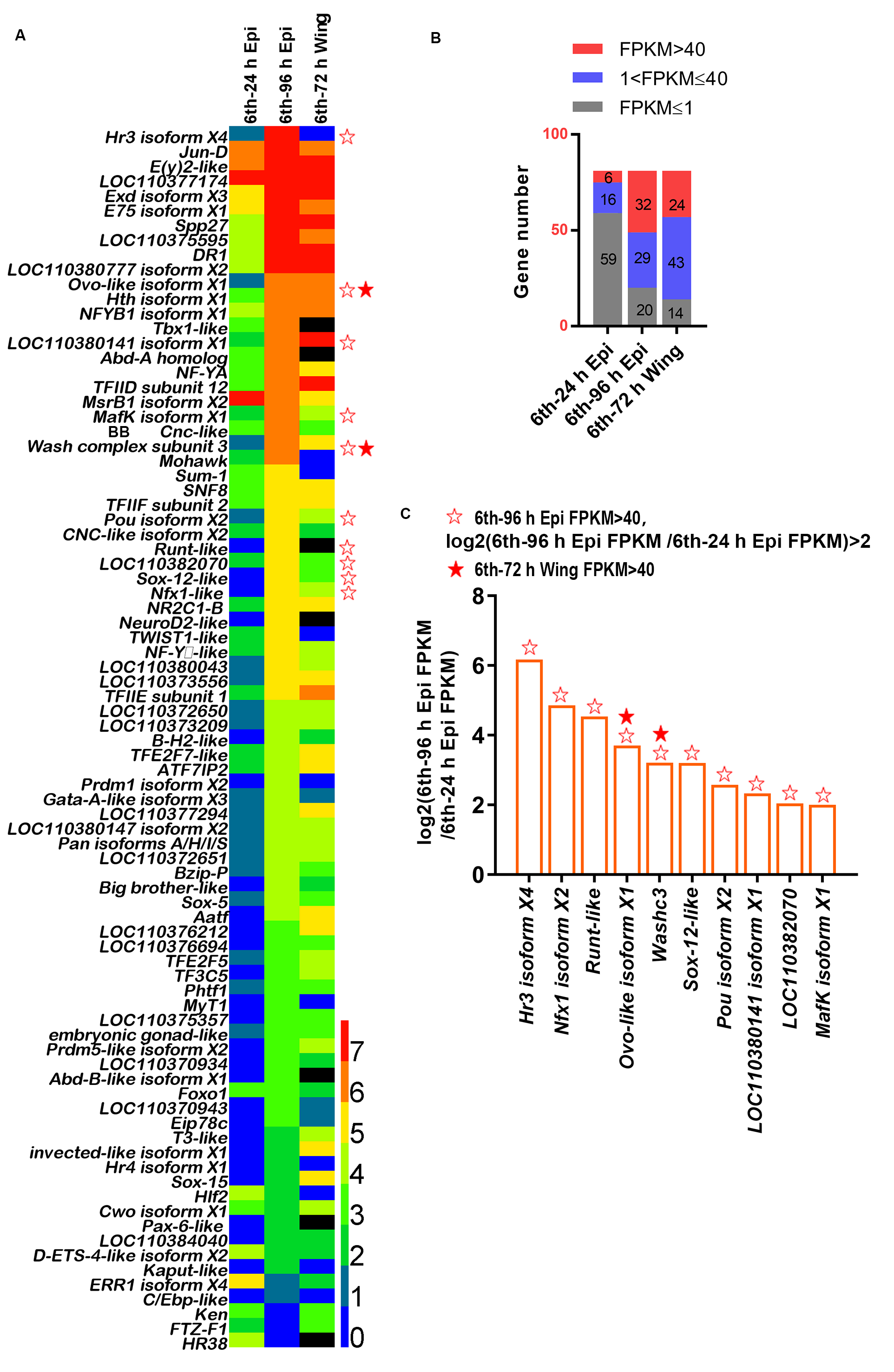

Supplement: S4 Fig — (A) A Hemi heatmap was used to analyze the expression of all transcription factors in the 6th-24 h epidermis, 6th-96 h epidermis and 6th-72 h wings. (B) FPKM value analysis of transcription factors in the 6th-24 h epidermis, 6th-96 h epidermis and 6th-72 h wing. The vertical axis represents the number of genes. (C) Transcription factors with upregulated expression in the 6th-96 h epidermis and FPKM values greater than 40 in the 6th-96 h epidermis and 6th-72 h wings. The vertical axis represents the value of log2 (6th-96 h Epi FPKM/6th-24 h Epi FPKM). The hollow stars represent seven transcription factors that are upregulated in the 6th-96 h epidermis and have FPKM greater than 40. The solid stars represent the transcription factors with FPKM values greater than 40 in the 6th-72 h wing of the ten transcription factors. (TIF) [file pgen.1011393.s004.tif]

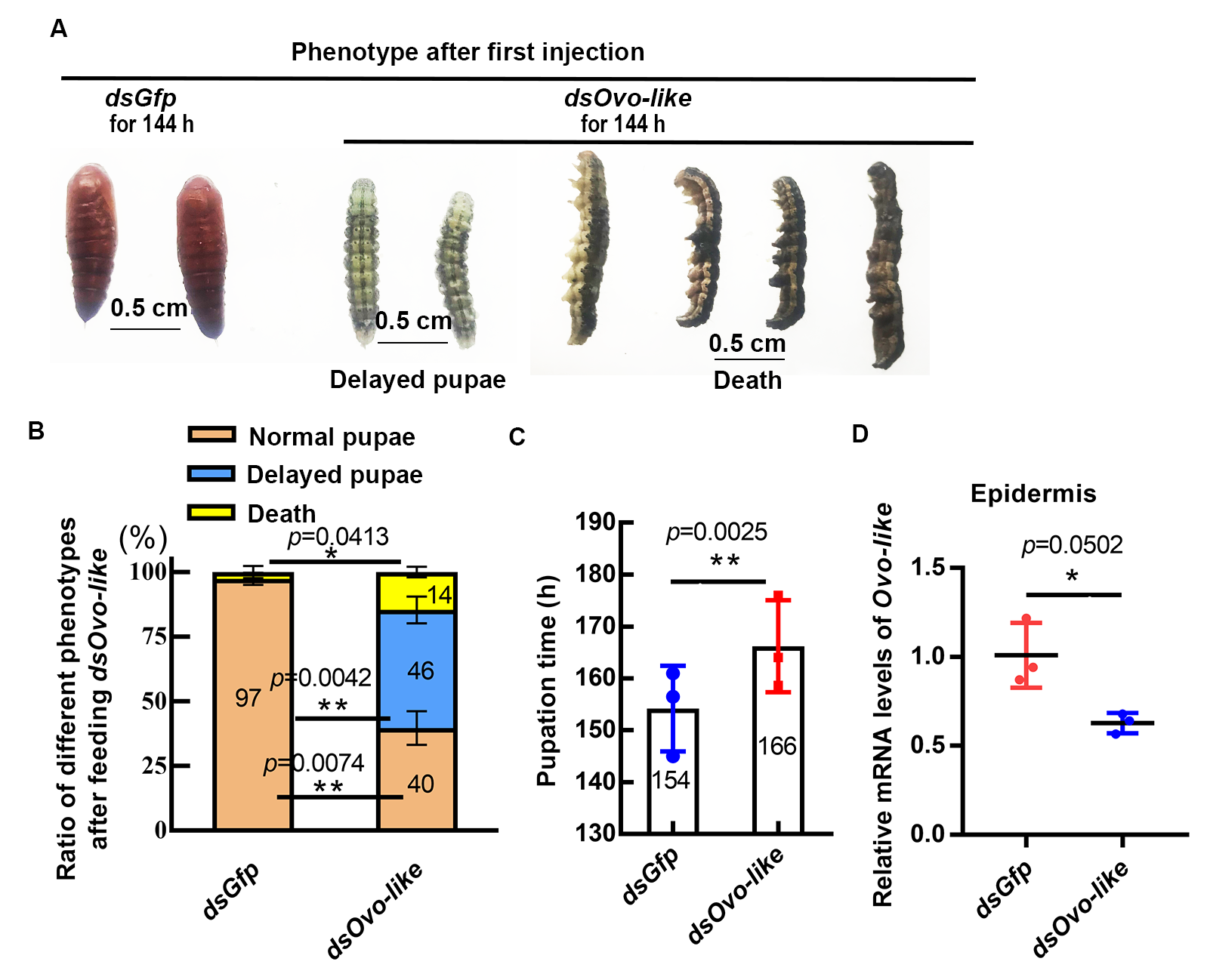

Supplement: S5 Fig — (A) Morphological differences after 144 h injection of dsOvo-like and dsGfp. Ruler 0.5 cm. (B) Statistical analysis of normal pupae, dead pupae, and delayed pupation in the dsOvo-like group and dsGfp group. Thirty larvae were used for each repetition. (C) Difference in larval pupation time after injection of dsOvo-like and dsGfp. (D) Epidermal RNA was extracted from 6th-96 h to detect the interference efficiency of dsOvo-like. All qRT-PCR experiments included three biological replicates and three technical replicates, error bars represent the mean ± SD, and a t test was used to analyze significant differences (*p<0.05, **p<0.01). (TIF) [file pgen.1011393.s005.tif]

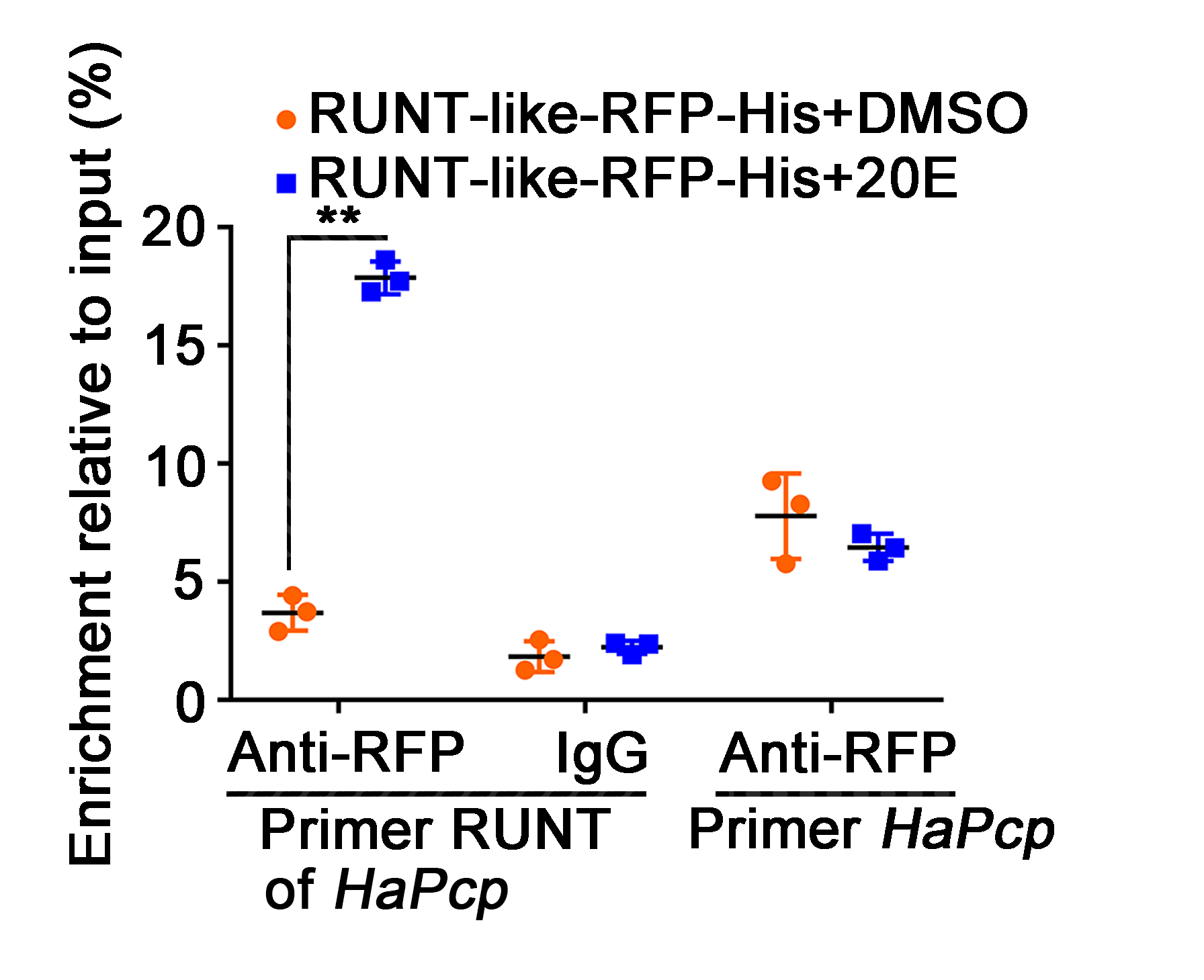

Supplement: S6 Fig — The primer RUNT of HaPcp is the HaPcp promoter sequence containing the RUNT-like binding site. The primer HaPcp, as a non-RUNT-like binding site control, targets the HaPcp open reading frame (ORF). IgG, nonspecific rabbit IgG. (TIF) [file pgen.1011393.s006.tif]

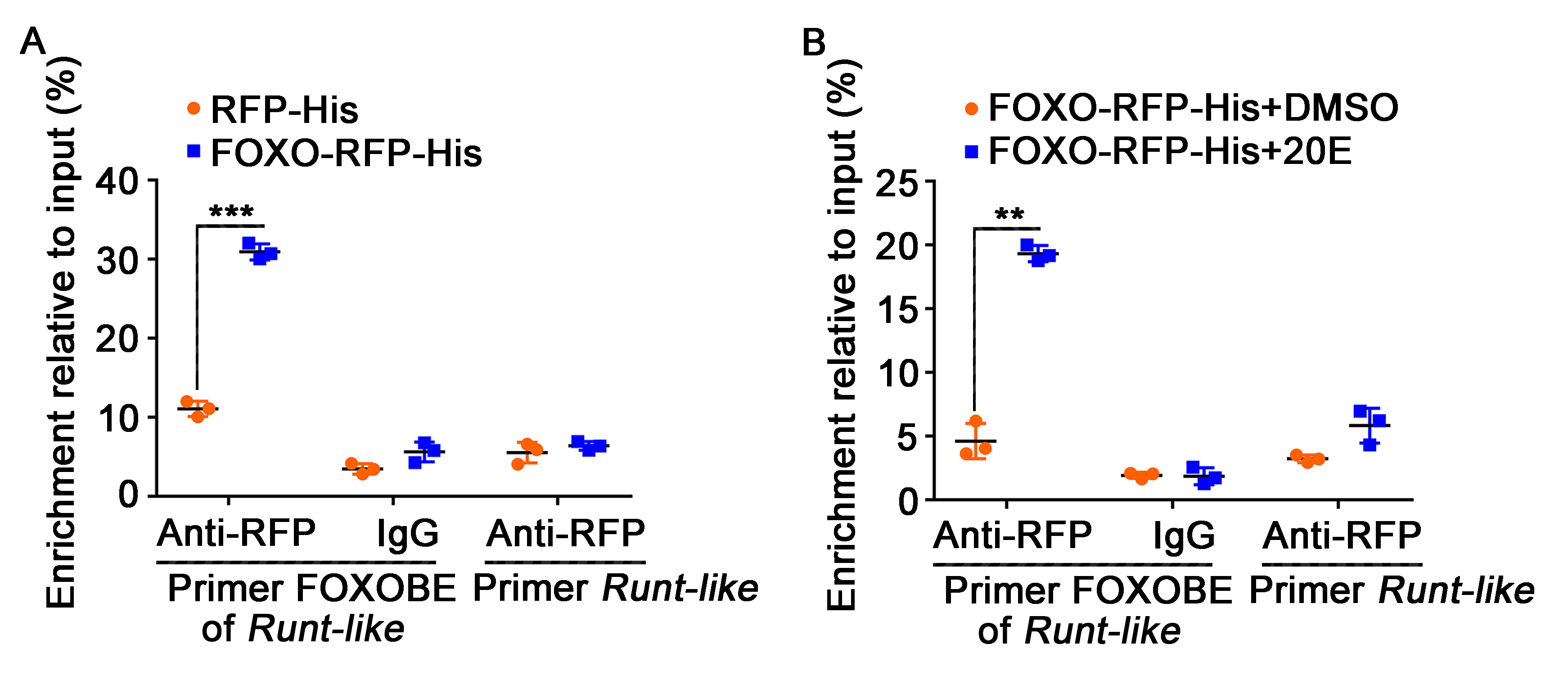

Supplement: S7 Fig — A. ChIP analysis of the FOXO binding site in the Runt-like promoter region. FOXO-RFP-His or RFP-His was overexpressed in HaEpi cells for 72 h. IgG, nonspecific rabbit IgG. Primer FOXOBE of Runt-like: primer targeted to the Runt-like promoter FOXOBE-containing sequence. Primer Runt-like: primer targeted to Runt-like ORF. B. ChIP experiment showing that 20E promoted Runt-like expression via FOXO binding to FOXOBE, as measured by qRT-PCR. The FOXOBE primer of Runt-like is the Runt-like promoter sequence containing FOXOBE. The primer Runt-like was used as a non-FOXOBE control targeting the Runt-like ORF. IgG, nonspecific rabbit IgG. (TIF) [file pgen.1011393.s007.tif]

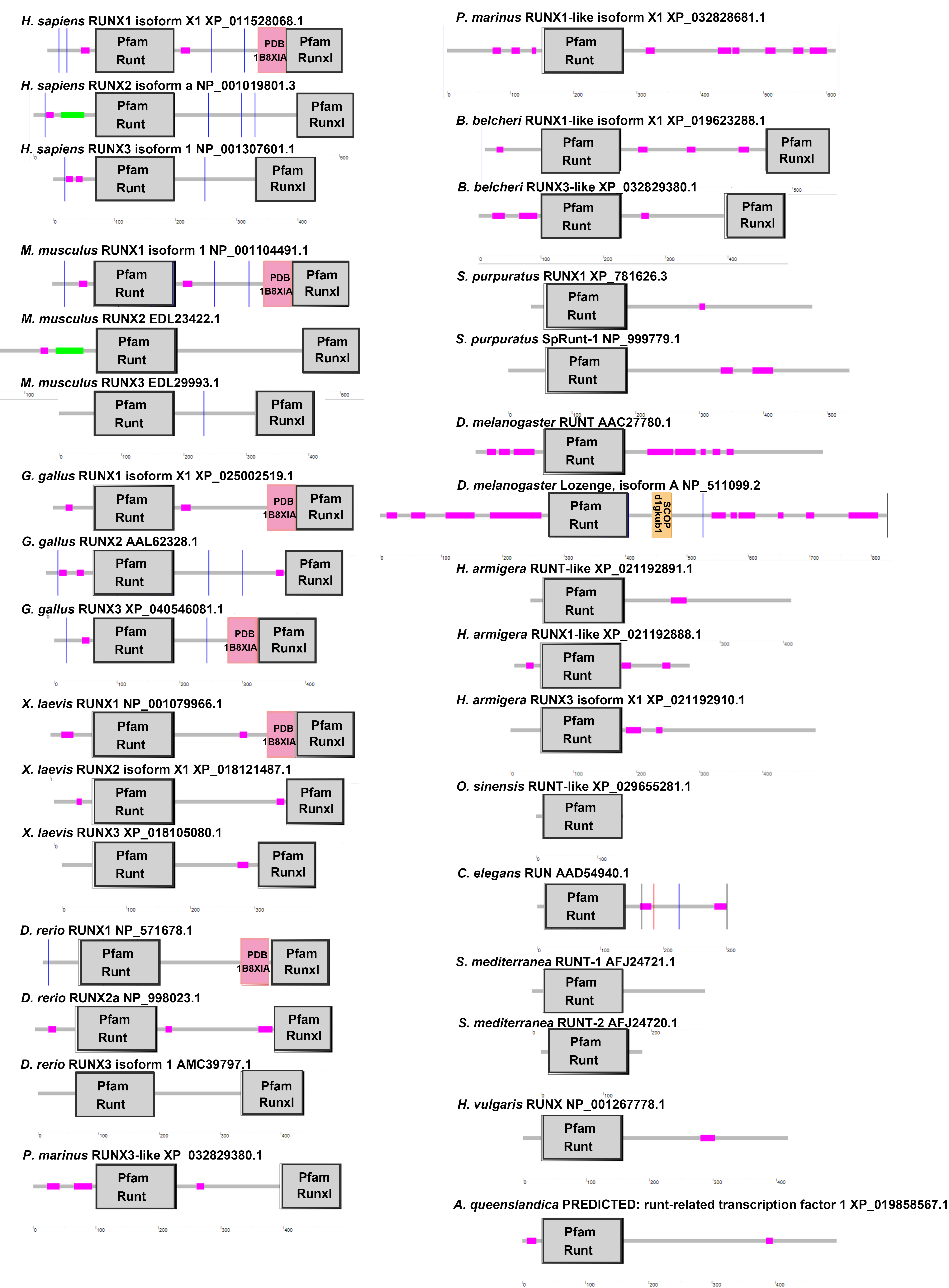

Supplement: S9 Fig — (TIF) [file pgen.1011393.s009.tif]
