# Supplementary material for: The transcription factor RUNT-like regulates pupal cuticle development via promoting a pupal cuticle protein transcription
Source: PLoS Genet. 2024 Sep 12;20(9):e1011393. doi: 10.1371/journal.pgen.1011393 (PMC11392391; doi:10.1371/journal.pgen.1011393)
Supplement: S1 Table — (DOCX) [file pgen.1011393.s010.docx]

**Table S1 cuticle protein**

| Nr | 6th-24 h Epi | 6th-96 h Epi | 6th-72 h Wing | log2(96/24) | Gene Symbol |
| --- | --- | --- | --- | --- | --- |
| Rpb1-like | 19.71 | 15515.16 | 450.72 | 8.36993 | LOC110373840 |
| Lcp-30-like | 148.45 | 8382.93 | 72.34 | 4.59293 | LOC110383810 |
| Col3a1-like | 90.92 | 797.72 | 8.34 | 1.863997 | LOC110378995 |
| LOC110384109 | 494.24 | 411.68 | 1.1 | -1.5284 | LOC110384109 |
| Extensin | 3.81 | 301.64 | 38.63 | 5.044061 | LOC110370275 |
| Cp10.9-like | 7.78 | 139.4 | 14.62 | 2.906009 | LOC110374745 |
| Cp16.8-like | 570.31 | 87.16 | 89.58 | -3.97226 | LOC110371697 |
| Hkr1-like | 0.23 | 87.04 | 0.13 | 7.298633 | LOC110375301 |
| Lcp16/17-like | 0.001 | 64.52 | 0 | 4.032752 | LOC110374827 |
| Lcp1 | 76557.29 | 48.49 | 218.53 | -11.8806 | LOC110384192 |
| LcpA2B-like | 1.36 | 46.92 | 3.85 | 3.849255 | LOC110376812 |
| Abd5-like | 539.45 | 44.36 | 14.52 | -4.85672 | LOC110384195 |
| Hrg-like | 0.001 | 30.44 | 27.95 | 4.025179 | LOC110374855 |
| Pcp52-like isoform X2 | 61.71 | 28.28 | 9.1 | -2.40671 | LOC110369631 |
| Cp18.6-like | 0.001 | 26.91 | 7.44 | 4.141824 | LOC110374829 |
| Lcp-14-like | 62344.27 | 17.85 | 111.98 | -13.0256 | LOC110372255 |
| SgAbd-2-like | 423.84 | 14.9 | 16.55 | -6.50208 | LOC110384200 |
| Flexible Cp12-like | 83602.64 | 11.7 | 0 | -14.6654 | LOC110382344 |
| SgAbd-5-like | 27045.25 | 10.78 | 1.88 | -12.5454 | LOC110384121 |
| Hccp66-like isoform X1 | 93.66 | 10.27 | 0.59 | -4.45973 | LOC110381235 |
| Flexible Cp12-like | 21.07 | 10.1 | 1.19 | -2.3256 | LOC110384126 |
| Lcp1-like | 10956.78 | 10.07 | 3.64 | -11.3375 | LOC110384086 |
| Nrg-P-like | 0.3 | 8.73 | 4.11 | 3.52482 | LOC110373845 |
| Lcp1-like | 23716.05 | 8.1 | 4.28 | -12.7717 | LOC110384084 |
| Lpcp-23-like | 127.21 | 7.82 | 20.26 | -5.28557 | LOC110369632 |
| Lcp-17-like | 35337.41 | 6.71 | 1.22 | -13.6197 | LOC110372245 |
| Pcp-like | 27.06 | 6.17 | 1.54 | -3.38626 | LOC110370887 |
| LCP-30-like | 13729.94 | 5.61 | 0 | -12.5204 | LOC110384186 |
| Lcp-17-like | 40334.91 | 4.43 | 1.68 | -14.4105 | LOC110383613 |
| Lcp-17-like | 40334.91 | 4.43 | 1.68 | -14.4105 | LOC110372244 |
| Lcp 1-like | 37564.55 | 3.25 | 1.56 | -14.8391 | LOC110384083 |
| Cp-1-like | 247.59 | 3.23 | 8.09 | -7.51707 | LOC110369633 |
| Lcp-17-like | 287.86 | 3.12 | 1.89 | -7.78711 | LOC110384169 |
| Hccp66-like | 90.72 | 2.5 | 0.28 | -6.43867 | LOC110381229 |
| DDB_G0274915-like | 218.83 | 2.37 | 3.6 | -7.79448 | LOC110376211 |
| Cp16.5-like isoform X1 | 16.65 | 1.9 | 0 | -4.4472 | LOC110381216 |
| Gpi-anchored 58 | 8.37 | 1.83 | 0.64 | -3.45817 | LOC110383464 |
| L3MBTL3 | 22.26 | 1.78 | 0.06 | -5.03853 | LOC110384173 |
| Cp38-like | 92.07 | 1.78 | 0 | -6.95488 | LOC110370226 |
| Lcp-22-like | 14439.75 | 1.59 | 0 | -14.4045 | LOC110384199 |
| Flexible Cp12-like | 15091.92 | 1.13 | 1.52 | -14.9524 | LOC110381314 |
| Abd-4-like | 50.66 | 1 | 0 | -6.92058 | LOC110384204 |
| Lcp-22-like | 21780.48 | 0.85 | 0.54 | -15.9411 | LOC110384203 |
| SgAbd-8-like | 120.37 | 0.6 | 0 | -8.90563 | LOC110384170 |
| Cp3-like | 116.41 | 0.59 | 0.72 | -8.89053 | LOC110376231 |
| Ascc2 | 17.36 | 0.47 | 0.14 | -6.46022 | LOC110384578 |
| Cp19-like | 23.58 | 0.45 | 0 | -6.96453 | LOC110374836 |
| SgAbd-2-like | 260.99 | 0.36 | 0.86 | -10.7657 | LOC110384198 |
| Pcp-like | 35.57 | 0.36 | 0.86 | -7.89053 | LOC110370919 |
| Abd-4-like | 22.58 | 0.13 | 2.15 | -7.89053 | LOC110384127 |
| Extensin-like | 8.54 | 0.12 | 0.29 | -7.38676 | LOC110384075 |
| LOC110384197 | 47.43 | 0.001 | 0 | -6.77881 | LOC110384197 |
| Cp65 | 66.04 | 0.001 | 0 | -4.90528 | LOC110377478 |
| Cp16.5-like | 20.54 | 0.001 | 0 | -4.28379 | LOC110377479 |
