# Supplementary material for: The transcription factor RUNT-like regulates pupal cuticle development via promoting a pupal cuticle protein transcription
Source: PLoS Genet. 2024 Sep 12;20(9):e1011393. doi: 10.1371/journal.pgen.1011393 (PMC11392391; doi:10.1371/journal.pgen.1011393)
Supplement: S2 Table — (DOCX) [file pgen.1011393.s011.docx]

**Table S2 Transcription factors**

| Nr | 6th-24 h Epi | 6th-96 h Epi | 6th-72 h Wing | log2(96/24) |
| --- | --- | --- | --- | --- |
| Hr3 isoform X4 | 3.66 | 516.64 | 0.59 | 6.167830864 |
| Jun-D | 82.27 | 420.17 | 105.13 | 1.090376324 |
| E(y)2-like | 89.52 | 386.68 | 406.37 | 1.097898875 |
| LOC110377174 | 478.37 | 307.01 | 207.67 | -1.939364468 |
| Exd isoform X3 | 45.91 | 227.73 | 265.22 | 1.077960882 |
| E75 isoform X1 | 37.64 | 225.12 | 120.47 | 1.381167056 |
| Spp27 | 22.52 | 152.46 | 194.08 | 1.497572923 |
| LOC110375595 | 25.1 | 130.05 | 85.1 | 1.110472259 |
| DR1 | 25.01 | 130.01 | 147.3 | 1.117622537 |
| LOC110380777 isoform X2 | 23.33 | 127.98 | 372.87 | 1.195155992 |
| Ovo-like isoform X1 | 3.75 | 117.03 | 72.72 | 3.704328439 |
| Hth isoform X1 | 15.19 | 106.01 | 83.37 | 1.612844107 |
| NFYB1 isoform X1 | 21.33 | 103.07 | 109.04 | 1.024158182 |
| Tbx1-like | 14.99 | 96.16 | 0 | 1.417888537 |
| LOC110380141 isoform X1 | 7.47 | 89.96 | 392.77 | 2.328157161 |
| Abd-A homolog | 9.15 | 80.83 | 0 | 1.878525531 |
| NF-YA | 13.3 | 78.93 | 56.69 | 1.35822107 |
| TFIID subunit 12 | 8.6 | 77.76 | 143.52 | 1.949135049 |
| MsrB1 isoform X2 | 207.46 | 77.42 | 32.28 | -2.464506088 |
| MafK isoform X1 | 7.36 | 70.43 | 27.49 | 2.00125778 |
| Cnc-like | 11.56 | 68.53 | 8.92 | 1.356301556 |
| Wash complex subunit 3 | 2.96 | 66.49 | 62.57 | 3.209220803 |
| Mohawk | 7.2 | 65.3 | 0.99 | 1.817193477 |
| Sum-1 | 11.51 | 62.06 | 0.24 | 1.169432999 |
| SNF8 | 11.29 | 55.08 | 41.04 | 1.023567942 |
| TFIIF subunit 2 | 9.21 | 53.09 | 36.69 | 1.264292186 |
| Pou isoform X2 | 3.66 | 52.54 | 17.64 | 2.578566708 |
| CNC-like isoform X2 | 6.31 | 51.59 | 4.75 | 1.840065515 |
| Runt-like | 0.86 | 48.13 | 0 | 4.53731068 |
| LOC110382070 | 4.43 | 43.89 | 7.97 | 2.04468006 |
| Sox-12-like | 1.97 | 43.53 | 15.53 | 3.202891641 |
| Nfx1-like | 0.8 | 41.68 | 21.85 | 4.854518082 |
| NR2C1-B | 5.03 | 38.7 | 41.22 | 1.646341273 |
| NeuroD2-like | 0.001 | 38.64 | 0 | 3.830362942 |
| TWIST1-like | 4.63 | 38.56 | 0.29 | 1.794806902 |
| NF-Yγ-like | 6.57 | 33.44 | 21.54 | 1.060523729 |
| LOC110380043 | 3.8 | 33.18 | 19.28 | 1.86519623 |
| LOC110373556 | 2.68 | 33.07 | 50.51 | 2.364896127 |
| TFIIE subunit 1 | 5.95 | 31.82 | 66.39 | 1.155635122 |
| LOC110372650 | 3.71 | 28.82 | 22.42 | 1.692791945 |
| LOC110373209 | 3.91 | 27.61 | 24.94 | 1.556318795 |
| B-H2-like | 1.23 | 27.21 | 5.88 | 3.202891641 |
| TFE2F7-like | 4.74 | 27.13 | 46.53 | 1.254642016 |
| ATF7IP2 | 5.31 | 26.43 | 40.85 | 1.002631121 |
| Prdm1 isoform X2 | 0.08 | 25.64 | 1.52 | 7.12889106 |
| Gata-A-like isoform X3 | 3.83 | 25.45 | 2.47 | 1.598613676 |
| LOC110377294 | 3.1 | 23.89 | 32.23 | 1.684954234 |
| LOC110380147 isoform X2 | 2.56 | 21.19 | 19.49 | 1.789753675 |
| Pan isoforms A/H/I/S | 3.19 | 20.61 | 22.26 | 1.346441227 |
| LOC110372651 | 3.76 | 19.8 | 17.55 | 1.133505578 |
| Bzip-P | 3.37 | 18.94 | 14.64 | 1.227081107 |
| Big brother-like | 1.78 | 18.84 | 7.06 | 2.138761304 |
| Sox-5 | 3.08 | 17.19 | 9 | 1.275082775 |
| Aatf | 1.49 | 16.03 | 53.91 | 2.163925227 |
| LOC110376212 | 1.19 | 15.51 | 34.8 | 2.440142021 |
| LOC110376694 | 1.89 | 15.51 | 14.6 | 1.789753675 |
| TFE2F5 | 2.39 | 15.11 | 29.89 | 1.397186456 |
| TF3C5 | 1.31 | 13.96 | 29.49 | 2.151817456 |
| Phtf1 | 2.55 | 13.61 | 14.2 | 1.109061069 |
| MyT1 | 1.23 | 13.45 | 1.12 | 2.116734997 |
| LOC110375357 | 1.34 | 11.85 | 9.65 | 1.885780562 |
| embryonic gonad-like | 2.16 | 10.48 | 11.17 | 1.03688169 |
| Prdm5-like isoform X2 | 1.59 | 9.3 | 16.37 | 1.287375799 |
| LOC110370934 | 0.39 | 8.42 | 4.62 | 3.160456375 |
| Abd-B-like isoform X1 | 0.81 | 8.06 | 0 | 2.171182781 |
| Foxo1 | 15.25 | 8.04 | 6.66 | -2.022934818 |
| LOC110370943 | 0.9 | 7.97 | 2.71 | 1.732441021 |
| Eip78c | 0.001 | 7.94 | 2.34 | 3.532139108 |
| T3-like | 0.22 | 7.24 | 24.59 | 4.420796671 |
| invected-like isoform X1 | 1.39 | 7.12 | 34.41 | 1.00125778 |
| Hr4 isoform X1 | 0.001 | 7.1 | 0.23 | 4.155763229 |
| Sox-15 | 0.62 | 6.64 | 55.05 | 2.244114304 |
| Hlf2 | 16.38 | 6.51 | 0.95 | -2.446201197 |
| Cwo isoform X1 | 13.43 | 6.38 | 18.76 | -2.169346297 |
| Pax-6-like | 0.86 | 6.27 | 0 | 1.596867525 |
| LOC110384040 | 0.92 | 6.27 | 7.86 | 1.500359544 |
| D-ETS-4-like isoform X2 | 20.1 | 5.48 | 4.27 | -3.118439188 |
| Kaput-like | 0.45 | 4.72 | 1.52 | 2.408682095 |
| ERR1 isoform X4 | 44.11 | 2.7 | 6.74 | -5.688471458 |
| C/Ebp-like | 0.1 | 2.62 | 0.27 | 2.444864431 |
| Ken | 8.18 | 1.58 | 14.12 | -3.021110033 |
| FTZ-F1 | 5.16 | 0.76 | 12.02 | -3.784951333 |
| HR38 | 19.89 | 0.47 | 0 | -6.662138596 |
