# Supplementary material for: The transcription factor RUNT-like regulates pupal cuticle development via promoting a pupal cuticle protein transcription
Source: PLoS Genet. 2024 Sep 12;20(9):e1011393. doi: 10.1371/journal.pgen.1011393 (PMC11392391; doi:10.1371/journal.pgen.1011393)
Supplement: S3 Table — (DOCX) [file pgen.1011393.s012.docx]

**Table S3 Primers used in the experiments.**

| Primer name | Sequence (5’-3’) |
| --- | --- |
| Exp-LC3-F | tactcaggatccatgaaattccaatata |
| Exp- LC3-R | tactcagagctcatatccatatacattc |
| Hr3-RTF | aagttctggtgaagagtttggc |
| Hr3-RTR | cttggagacatcctgtggcttt |
| Neurod2- RTF | gaacggagcggggcaag |
| Neurod2- RTR | tcgggcatggagtgacg |
| Runt-like- RTF | gggaagtcgttctcgctga |
| Runt-like- RTR | ttagtcctgggctctctggg |
| Ovo- RTF | atgctctctggaatcccactgc |
| Ovo- RTR | tggcgagtaggggtgctgttt |
| Washc3- RTF | ggcgacccaaatgactactct |
| Washc3- RTR | ggtcccagtaaccatcgtctt |
| Rpb1-like- RTF | catcttggcgttctcactcg |
| Rpb1-like- RTR | acaggctctttaggaggcaac |
| Hkr1-like- RTF | gggtgctcacttcgttcca |
| Hkr1-like- RTR | atgttgggatacggctggt |
| Extensin- RTF | aagtccgttgatgcttctccac |
| Extensin- RTR | atcgttttggggttcggc |
| Lcp30-like- RTF | ggaccttacacccacgactacg |
| Lcp30-like- RTR | acagccttctcttcagcacgga |
| Runt-like - RNAiF | gcgtaatacgactcactatagcctacctcaactatgcgt |
| Runt-like- RNAiR | gcgtaatacgactcactataggtcttgtgggatggaaa |
| Ovo- RNAiF | gcgtaatacgactcactataggtgatggaaagaaagaat |
| Ovo- RNAiR | gcgtaatacgactcactatagagaagggaggaagactg |
| Rpb1-like- RNAiF | gcgtaatacgactcactatagactttggggataatggatac |
| Rpb1-like- RNAiR  Op-Runt-like-GFP- F  Op-Runt-like-GFP- R | gcgtaatacgactcactatagggttgggtagaagatttgag  tcgttaacacgtcaagagctcatgcacctcccgcacgcg  cttcgaaccggtaccgtcgacatatggccgccaaactgactt |

**GenBank corresponding to the genes in the table.** *Runt-like*: XP_021192891.1; *Lc3*: [XP_021181573.1](https://www.ncbi.nlm.nih.gov/projects/sviewer/sequence.cgi?netcache=0&id=gi|1199374729&format=fasta&filename=XP_021181573.1.fa&ranges=0-116); *Hr3*: XP_021186797.1; *Neurod2*: XP_021184401.1; *Ovo*:XP_021183867.1; *Washc3*: XP_021188673.1; *Rpb1-like*: XP_021186897.1; *Hkr1-like*: XP_021189040.1; *Extensin*: XP_021181695.1; *Lcp30-like*: XP_021200394.1**.**
