# Supplementary material for: The transcription factor RUNT-like regulates pupal cuticle development via promoting a pupal cuticle protein transcription
Source: PLoS Genet. 2024 Sep 12;20(9):e1011393. doi: 10.1371/journal.pgen.1011393 (PMC11392391; doi:10.1371/journal.pgen.1011393)
Supplement: S1 Data — (PDF) [file pgen.1011393.s013.pdf]

Fig 2A

HaPap

	Epidermis			Wing		
6th-24 h	0.571701	1.208597	0.470848	0.658231	1.316463	0.498846
6th-96 h	6.868523	9.713559	11.23556	12.23832	10.07937	11.49818
	t-test: 0.0221			t-test: 0.0067		

Fig 2B

Hkr1-like

	Epidermis			Wing		
6th-24 h	0.968171	1.39797	0.73884	1.011619	1.211393	0.816014
6th-96 h	28198.55	41572.25	43337.65	0.004466	0.005853	0.004688
	t-test: 0.0157					

Fig 2C

Extensin

	Epidermis			Wing		
6th-24 h	0.866537	1.023374	1.127661	0.697372	0.946058	1.515717
6th-96 h	1534.266	1513.144	2230.781	26.17287	29.65082	19.29293
	t-test: 0.0175			t-test: 0.0178		

Fig 2D

LCP-30-like

	Epidermis			Wing		
6th-24 h	0.874583	1.211393	0.943874	0.816014	1.011619	1.211393
6th-96 h	41.16463	44.42603	35.34253	4.648182	3.64689	4.489848
	t-test: 0.0043			t-test: 0.0111		

Fig 2E

LCP16/17-like

	Epidermis			Wing		
6th-24 h	1.127661	0.981686	0.903335	0.876606	1.06437	1.071773
6th-96 h	68.75217	69.71191	42.32192	0.44648	0.452712	0.491978
	t-test: 0.0219					

Fig 3A

HaPap

	Epidermis			Midgut			Fat body		
5F	1.670176	0.641713	0.933033	0.0000118	0.0000118	0.0000118	0.001529	0.001233	0.001708
5M	1.36604	3.160165	1.777685	0.00000503	0.00000353	0.00000543	0.000437	0.001137	0.00074
6th-6 h	0.882703	0.368567	0.400535	0.000139	0.0000471	0.0000666	0.001143	0.001605	0.000526
6th-24 h	0.571701	1.208597	0.470848	0.0000206	0.0000185	0.0000222	0.00012	0.000151	0.000186
6th-48 h	0.326842	0.012988	0.015233	0.0000356	0.0000281	0.0000121	0.00012	0.000151	0.000186
6th-72 h	0.766664	1.460707	0.705475	0.000242	0.000307	0.000229	0.000202	0.000192	0.0002
6th-96 h	6.868523	4.658934	11.23556	0.047257	0.027331	0.084008	0.000275	0.000366	0.000732
6th-120 h	6.868523	4.658934	11.23556	0.001808	0.013286	0.015398	0.231112	0.234339	0.388683
P-2 d	10.48315	7.516182	9.253505	0.010922	0.017498	0.012287	1.440597	1.914101	1.084227
P-4 d	3.672256	2.725775	3.828201	0.002064	0.002108	0.002108	0.509328	0.468677	0.40239
P-6 d	3.474166	4.584189	3.775497	0.164938	0.164938	0.142595	0.218646	0.150378	0.110083
P-8 d	15.70697	14.55389	14.96306	0.136787	0.019104	0.051119	0.00411	0.002264	0.002041

	Wing			Brain		
5F				5.438969	3.613341	3.819366
5M				0.673617	0.692555	0.417544
6th-6 h				0.034118	0.018928	0.023303
6th-24 h	0.658231	1.316463	0.498846	0.110083	0.139339	0.235969
6th-48 h	4.789915	7.110741	5.938094	0.071463	0.092997	0.113702
6th-72 h	16.52601	17.71214	10.10268	0.036991	0.013824	0.011229
6th-96 h	12.23832	6.204579	11.49818	2.302711	4.637455	1.427344
6th-120 h	10.72818	10.29116	12.84677	1.725084	3.801758	1.666321
P-2 d	29.04061	33.12848	9.781122	0.048361	0.033726	0.010309
P-4 d	44.3235	57.68003	34.77552	0.015625	0.006524	0.011842
P-6 d	5.302478	2.632925	7.871653	0.001394	0.00348	0.000566
P-8 d	5.217408	3.123868	3.954056	0.001586	0.000735	0.005119

Fig 3B

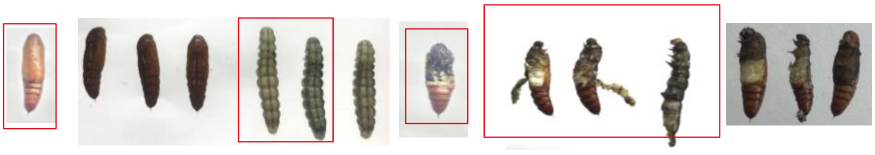

dsGFP *dsHaPap*

Fig 3C

	Normal pupae			Delayed pupae			Death		
	1st	2nd	3rd	1st	2nd	3rd	1st	2nd	3rd
dsGFP	100	96	100	0	0	0	0	4	0
dsHaPap	35	27	32	49	40	42	16	33	26
	t-test: 0.0003			t-test: 0.0039			t-test: 0.0265		

Fig 3D

dsGFP	*dsHaPap*
162.947	186
170	185
168	192
t-test:	0.0185

Fig 3E

dsGFP	*dsHaPap*
0.7457	0.043485
1.138131	0.043485
1.178267	0.043485
t-test:	0.0194

Fig 3F

dsGFP *dsHaPap*

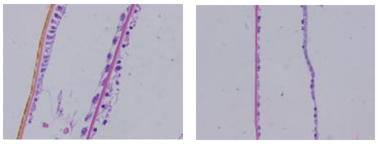

Fig 3G

dsGFP *dsHaPap*

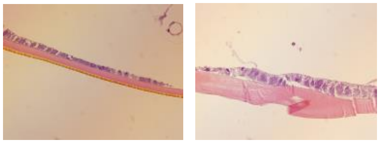

Fig 3H

dsGFP *dsHaPap*

6th-48 h

6th-72 h

6th-96 h

6th-120 h

6th-144 h

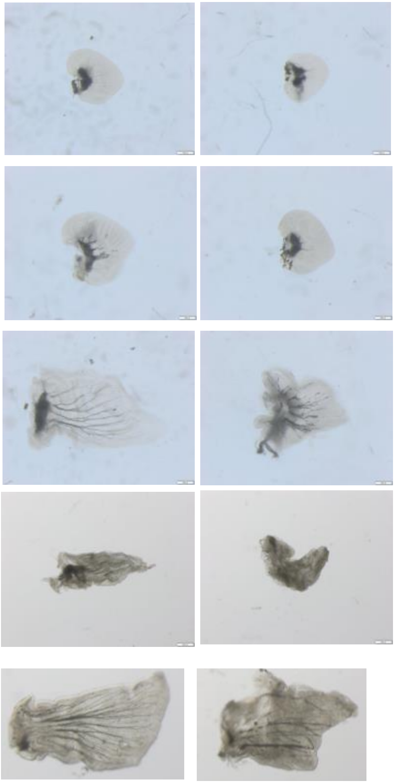

Fig 4A

Hr3

	Epidermis			Wing		
6th-24 h	0.737135	0.737135	1.484524	1.414214	0.707107	1
6th-96 h	2.83497	2.502436	3.324952	45.25483	42.22425	50.91433
	t-test: 0.0063			t-test: 0.0031		

Fig 4B

Nfx1

	Epidermis			Wing		
6th-24 h	1.3692	1.277509	0.571701	1.140764	0.913831	0.959264
6th-96 h	1.089249	1.112136	1.059463	0.026645	0.009552	0.024689
				t-test: 0.0044		

Fig4C

Runt-like

	Epidermis			Wing		
6th-24 h	6.964405	15.88948	3.605002	4.8121	6.573523	5.376498
6th-96 h	101.3592	114.8282	133.7443	21.40684	15.03236	15.77972
	t-test: 0.0107			t-test: 0.0405		

Fig 4D

Ovo-like

	Epidermis			Wing		
6th-24 h	4.981798	0.631418	0.317905	1.117287	1.125058	0.795536
6th-96 h	27.09585	17.63048	26.53823	31.85247	33.90282	21.01479
	t-test: 0.0146			t-test: 0.0188		

Fig 4E

Washc3

	Epidermis			Wing		
6th-24 h	1.313425	0.484085	1.572798	0.988514	1.030492	0.981686
6th-96 h	27.66519	34.5353	50.56264	1.922966	1.388313	1.135504
	t-test: 0.0316					

Fig 5A

Runt-like

	Epidermis			Midgut			Fat body		
5F	0.668964	0.823591	1.815038	0.854607	0.121863	0.802923	0.968171	0.675175	4.510644
5M	16.99062	4.489848	3.264058	0.248273	0.087171	0.25	13.29981	7.029068	24.13966
6th-6 h	6.468061	11.49818	3.663781	0.203533	6.291192	0.289841	2.351096	3.899619	2.383916
6th-24 h	6.964405	15.88948	3.605002	0.66281	0.138376	0.075189	23.86239	0.93088	3.723519
6th-48 h	4.356995	5.039684	3.740765	0.220166	0.202594	0.211198	4.150639	2.19365	27.53765
6th-72 h	34.2176	90.30079	62.97323	0.184284	0.307786	14.22148	5.241574	6.233317	8.876556
6th-96 h	101.3592	114.8282	133.7443	0.312083	0.203063	0.346277	3.723519	9.6242	5.265851
6th-120 h	402.6363	355.4082	157.951	1.109569	0.320856	0.840896	21.9072	8.897089	10.58048
P-2 d	351.326	323.2859	429.5454	1.927414	3.045474	5.121856	29.446	17.83518	24.08395
P-4 d	78.9755	107.8837	181.4381	10.72818	18.93959	8.359017	19.97329	27.47409	19.69831
P-6 d	722.4063	266.2553	655.5976	13.29981	8.186991	14.15592	5.993228	3.038445	3.017457
P-8 d	276.9215	313.7199	397.0931	8.693879	38.85424	19.56224	5.01645	6.090947	3.828201

	Wing			Brain		
5F				41.35529	34.05985	47.50475
5M				14.79119	11.60494	51.15015
6th-6 h				12.84677	30.13427	17.79418
6th-24 h	4.8121	6.573523	5.376498	7.691864	2.7007	16.14856
6th-48 h	6.900336	4.8121	9.963597	20.67765	29.04061	6.821079
6th-72 h	13.29981	29.31025	2.854689	8.574188	20.25211	6.543216
6th-96 h	21.40684	15.03236	15.77972	5.775717	4.500234	0.473029
6th-120 h	40.03898	24.99094	44.11916	5.829343	10.8028	6.789632
P-2 d	20.67765	25.10669	58.89201	19.38228	17.14838	9.105048
P-4 d	89.67703	128.5929	108.8854	6.438241	8.673815	7.498836
P-6 d	20.91791	12.43786	25.57508	0.528509	3.863745	1.231144
P-8 d	11.41875	7.963117	5.476801	2.66968	8.092956	1.401204

Fig 5B

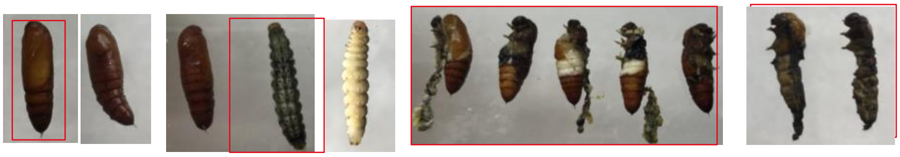

dsGFP *dsRunt-like*

Fig 5C

	Normal pupae			Delayed pupae			Death		
	1st	2nd	3rd	1st	2nd	3rd	1st	2nd	3rd
dsGFP	96	100	96	0	0	0	4	0	4
dsRunt-like	16	18	24	46	42	40	38	40	36
	t-test: 0.0046			t-test: 0.0017			t-test: 0.0015		

Fig 5D

dsGFP	*dsRunt-like*
150	162
132	150
139	164
t-test:	0.0395

Fig 5E

dsGFP	*dsRunt-like*		
	Runt-like	*RunX1*	*RunX3*
1.41095	0.166855	1.099362	1.414214
0.844791	0.20829	0.92445	1.006956
0.838956	0.063226	0.983957	0.702222
t-test:	0.0404		

Fig 5F

dsGFP *dsRunt-like*

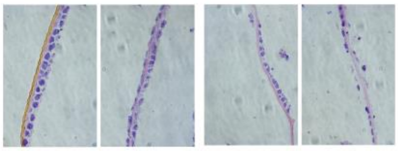

Fig 5G

dsGFP *dsRunt-like*

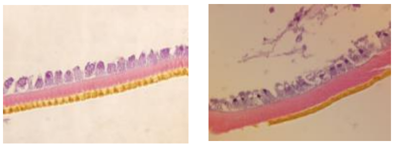

Fig 5H

dsGFP *dsRunt-like*

6th-48 h

6th-72 h

6th-96 h

6th-120 h

6th-144 h

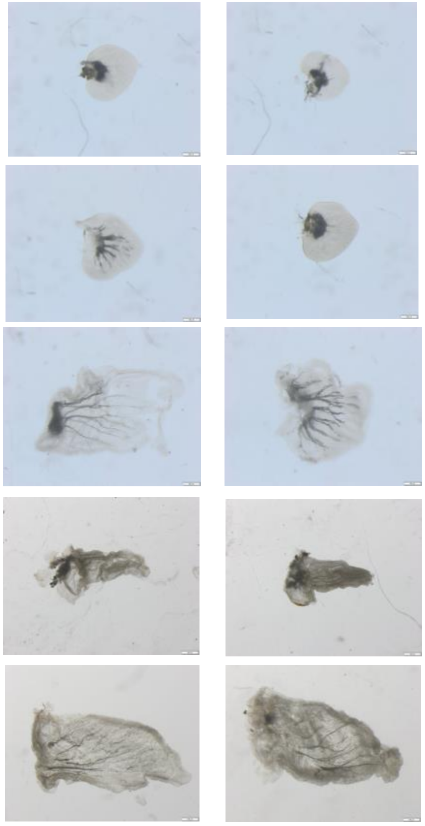

Fig 6A

	dsGFP			*dsRunt-like*			
HaPap	1.016305	1.059463	0.928731	0.086569	0.086569	0.192109	t-test: 0.0067
Extensin	1.076738	1.236847	0.750886	0.136156	0.08983	0.093644	t-test: 0.0232
Lcp-30-like	1.404445	1.214195	0.586417	0.740549	0.545884	0.676737	

Fig 6C

LUCI-GFP-His/β-Actin				
RFP-His	-	-	+	+
RUNT-like-RFP-His	+	+	-	-
pHaPcp-LUCI-GFP-His	+	+	+	+
DMSO	+	-	+	-
20E (2 µM)	-	+	-	+
	0.291391	1.906797	0.134377	0.079401
	0.106416	1.398064	0.351513	0.229175
	0.196814	1.49984	0.393422	0.181484
t-test:		0.0057		

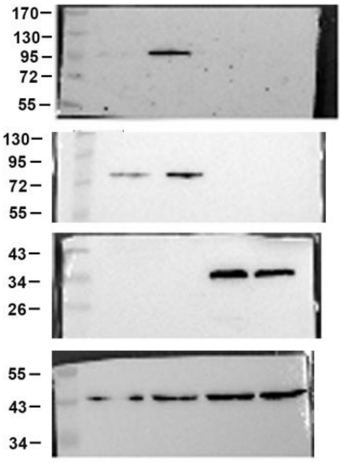

Fig 6D

Relative luciferase activity of *HaPcp* promoter				
RFP-His	-	-	+	+
RUNT-like-RFP-His	+	+	-	-
pHaPcp-LUCI-GFP-His	+	+	+	+
DMSO	+	-	+	-
20E (2 µM)	-	+	-	+
	1.104	2.0833	1.1875	1.1818
	1.21875	2.666666	1.0638	1.060606
	1.1212	2.421	1.1818	1.15909
t-test:		0.0122		

Fig 7A

Runt-like

DMSO	100ng	200ng	300ng	500ng
0.752623	2.297397	28.18131	11.00433	2.214018
0.806642	2.549121	27.98665	8.574188	3.332643
1.647182	2.531513	28.97359	5.314743	3.450168
t-test:	0.0331	0.0018	0.0078	0.0254

Fig 7B

Runt-like

DMSO	1 h	DMSO	3 h	DMSO	6 h	DMSO	12 h	DMSO	24 h
0.974905	1.194715	1.081725	1.228303	1.353474	4.723971	1.205808	48.95327	1.006956	26.47698
0.819794	1.632029	0.842842	1.262835	1.194715	4.563055	0.716978	45.67502	0.933033	16.07411
1.251218	1.620756	1.096825	1.689582	0.618423	4.85678	1.156688	42.6163	1.06437	15.74331
t-test:					0.0062		0.0017		0.0254

Fig 7C

Ecr				*Runt-like*			
DMSO	20E	*dsGfp*+20E	*dsEcR*+20E	DMSO	20E	*dsGfp*+20E	*dsEcR*+20E
0.788218	4.198867	4.500234	1.362888	2.445281	7.110741	8.594021	0.066986
0.9373545	4.756828	4.169863	2.051482	1.505247	4.69134	5.669939	0.022406
1.353474	3.329467	5.028053	1.33484	0.271684	5.578975	5.630774	0.046391
t-test: 0.0316		0.0231		0.0199		0.0210	

Fig 7D

Foxo				*Runt-like*			
DMSO	20E	*dsGfp*+20E	*dsFoxo*+20E	DMSO	20E	*dsGfp*+20E	ds*Foxo*+20E
1.146047	11	11.71269	1.580083	2.445281	7.110741	10.00975	0.487452
1.440597	11.41875	11.95879	1.021012	1.505247	4.69134	6.603969	0.371131
0.6056964	11.90366	12.99604	1.101905	0.271684	5.578975	11.26155	0.598739
t-test: 0.0316		0.0231		0.0199		0.0210	

Fig 8B

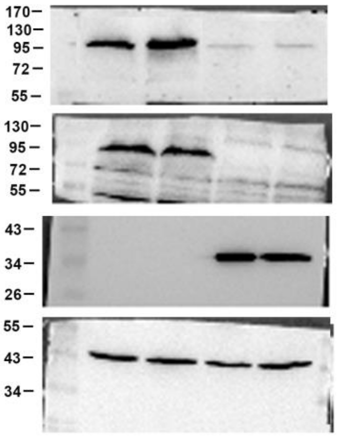

LUCI-GFP-His/β-Actin

RFP-His	-	-	+	+
FOXO-RFP-His	+	+	-	-
pRunt-like-LUCI-GFP-His	+	+	+	+
DMSO	+	-	+	-
20E (2 µM)	-	+	-	+
	0.693378	1.323033	0.308632	0.48727
	0.666677	1.424333	0.172032	0.140714
	0.834758	1.14187	0.099465	0.109187
t-test:		0.0281	0.0351	

Fig 8C

Relative luciferase activity of *HaPcp* promoter

RFP-His	-	-	+	+
FOXO-RFP-His	+	+	-	-
pRunt-like-LUCI-GFP-His	+	+	+	+
DMSO	+	-	+	-
20E (2 µM)	-	+	-	+
	4.106606	9.468833	1.1875	2.3818
	3.04833	9.250466	1.2938	2.360606
	3.99309	8.03728	1.1918	2.55909
t-test:	0.0143			
